# Barriers in operating proactive source reduction informed by an early warning system for dengue prevention: A qualitative study from end-user perspectives in Vietnam

**DOI:** 10.1371/journal.pntd.0014754

**Published:** 2026-09-25

**Authors:** Kien Quoc Do, Ha Thu Nguyen, Thi Thanh Thao Nguyen, Cong Tuan Pham, Nguyen T. T. K, Dang Khanh Linh Vien, Dung Phung

**Affiliations:** 1 Faculty of Health, Medicine and Behavioural Science, University of Queensland, Brisbane, Australia; 2 Department of Disease Control, Institute Pasteur in Ho Chi Minh City, Ho Chi Minh City, Vietnam; 3 Queensland Alliance for Environmental Health Sciences, University of Queensland, Brisbane, Australia; 4 Centre for Environment and Population Health, Griffith University, Brisbane, Queensland, Australia; 5 School of Preventive Medicine and Public Health, Hanoi Medical University, Hanoi, Vietnam; 6 Center for Disease Control, Ho Chi Minh City, Vietnam; The University of Sydney School of Veterinary Science, AUSTRALIA

## Abstract

**Background:**

Dengue is a major vector-borne disease, with rising incidence driven by climate change, rapid urbanization, and increased international travel. Although early warning systems (EWSs) have shown promising predictive performance in endemic settings, limited evidence on implementation barriers from local end users hinders their integration into routine proactive vector control.

**Methods:**

A qualitative study with in-depth interviews was conducted with local end users, including local authorities, health practitioners, and health volunteers. A deductive approach utilizing a predefined conceptual framework was employed to code the verbatim transcripts. Inductive coding was subsequently incorporated to capture emerging concepts that were not represented in the initial framework. Thematic analysis was performed to analyse the collected data. To enhance the methodological rigor, interviews and coding were performed by multiple and diverse researchers from various academic and practi**ce** institutions, and the transcripts were validated through member checking with selected participants.

**Results:**

The lack of supportive policies, limited engagement from relevant stakeholders due to low familiarity with EWS concepts, and operational challenges were identified as critical barriers to implementing EWS-informed proactive source reduction. The perception that dengue risks were only during the rainy season, together with socio-economic constraints, water scarcity exacerbated by extreme weather events, and the application of inappropriate source reduction measures, further constrained household participation. The multi-level barriers collectively disrupted the translation of EWS alerts into proactive dengue prevention.

**Conclusions:**

Early warning information alone is insufficient to translate into effective and sustained proactive vector control for dengue prevention. Strengthening enabling policies, local stakeholder and community engagement, and awareness of EWS-informed risk management across multiple levels are essential to translate EWS alerts into timely and sustained vector control actions in dengue-endemic settings similar to Vietnam. Further studies across diverse settings are needed to assess the broader applicability of EWS-informed approaches.

## Author summary

Dengue is increasing worldwide. Early warning systems (EWS) have been developed to predict outbreaks before they occur. These systems can help authorities take early action before the emergence of outbreaks. However, little is known about why these EWSs have not operated in dengue prevention. In this study, we interviewed local authorities, health officials, community representatives, and health volunteers in Vietnam to understand barriers to operating proactive larval control informed by EWS. We found that the absence of supportive policies, weak stakeholder engagement due to limited understanding of EWS concepts, and operational challenges related to local socio-economic features and water scarcity reduced the usefulness of EWS alerts. Community members often perceived dengue as a seasonal problem occurring only during the rainy season, which limited proactive action informed by EWS. Many also viewed proactive source reduction activities as primarily the responsibility of health authorities and local governments, rather than a shared community responsibility, further reducing household engagement in self-practising early preventive measures. Our findings suggest that technological tools alone are not enough. Proactive intervention informed by EWS must be supported by strong policies, effective risk communication, and context-appropriate control measures. These lessons are relevant for dengue-endemic countries like Vietnam, seeking to translate predictive tools into disease prevention.

## Introduction

Dengue is a major vector-borne disease transmitted through the bites of the *Aedes* mosquito, and poses a global public health concern due to its rapid increase in incidence and geographic expansion [1,2]. According to the World Health Organization (WHO), half of the world’s population is now living in dengue infection risk areas, and the disease has spread to more than 100 countries and territories worldwide [2]. In 2024 alone, an estimated 14.1 million cases were recorded globally, representing a 12-fold increase over the past decade [3]. Climate change, globalization, unplanned urbanization, and failures in vector control measures are key drivers of the global resurgence of dengue [1,4].

Vietnam is a dengue hyper-endemic country. The national dengue burden in Vietnam remains among the highest in Southeast Asian countries and is projected to account for 15% of dengue incidence in this region by 2080 [5,6]. In 2023 alone, more than 369,000 cases were reported nationwide, placing Vietnam among the top five countries globally in dengue burden [7]. Southern Vietnam is disproportionately affected, where climatic variability and increasing human mobility have intensified transmission dynamics [8–10].

Although dengue vaccine and the release of mosquitoes carrying *Wolbachia* have achieved promising results [11–13], concerns regarding their safety and cost-effectiveness restrict their large-scale rollout [14–16]. Consequently, vector control measures, particularly source reduction, remain the cornerstone of dengue control. However, current control strategies are largely reactive, relying on hospital-based surveillance data. Given that a substantial proportion of dengue infections are asymptomatic yet infectious [17,18], this reactive approach often results in delayed and suboptimal vector control.

Recent advances in early warning systems (EWS) have highlighted their potential capacity to enable proactive dengue prevention through timely, targeted intervention, and more efficient allocation of resources [8,19,20]. Even though EWSs have been piloted in several countries, none have yet been integrated into routine national dengue control operations [21]. While substantial research has focused on improving the predictive accuracy of dengue EWSs [8,19], far less attention has been paid to how decentralised health systems in endemic settings interpret and act upon probabilistic early warning signals. Integrating EWS into routine dengue control requires a shift from outbreak-driven response to EWS-informed proactive intervention, an operational transition that demands political endorsement, intersectoral coordination, resource mobilisation, and community engagement.

Empirical evidence on how health systems interpret and operationalise EWS outputs remains limited. This qualitative study explores barriers to implementing EWS-informed proactive source reduction in Vietnam. By examining perspectives across multiple levels of the local stakeholders, health system, and community through the expansion of the predefined conceptual frameworks. The study aims to generate empirical evidence to support the integration of EWS and to guide proactive interventions. The findings further provide transferable insights for other dengue-endemic countries with contexts that are similar to Vietnam that are seeking to operationalize EWS in dengue prevention.

## Methods

### Ethics statement

This study obtained ethics approvals from the University of Queensland (Ratification of external Human Research Ethics approval for project # 2023/HE001441) and the National Institute of Hygiene and Epidemiology in Vietnam (HĐĐĐ-07/2023). The study collected data from interviews; no identifiable information was recorded.

### Study settings

Dengue control in Vietnam is implemented consistently from the central to the local levels. The local level encompasses both provincial and commune tiers. The technical oversight is provided by the provincial Centre for Disease Control (CDC) and the Regional Health Centre (RHC). The RHC is responsible for coordinating and guiding dengue control activities for a group of communes. The community-based intervention is led by the Commune Health Stations (CHSs) through health volunteers (HVs), and supported administratively by Commune People’s Committees (CPCs) [22].

Dengue control in Vietnam is primarily reactive and triggered by index cases identified through routine surveillance [22]. Interventions include source reduction and outbreak response, with adulticide application within a 200-meter radius of index cases. Village-wide expansion is implemented when outbreak thresholds are exceeded, or when more than three outbreaks are reported within the same village in 2 consecutive weeks, or when the incidence in 2 consecutive weeks is greater than 150 cases/100,000 persons [22].

The study was conducted in An Giang and Tay Ninh provinces in southern Vietnam, which have a high dengue burden, with annual reported cases between 2021 and 2025 being 8,377 in An Giang and 10,713 in Tay Ninh (S1 Table) [23]. In 2025, Tay Ninh experienced a large, province-wide dengue outbreak. Dengue transmission in these provinces is marked by seasonal transmission and is consistent with dengue patterns in tropical countries, with the increase during the rainy season commencing from June to November [10]. Similar to other provinces in southern Vietnam, household water storage is commonly practiced in the study provinces because of the concerns regarding limited water supply and preferences for rainwater storage based on perceptions of lower cost, water quality, and supply security for daily use [24].

E-Dengue is an EWS that is capable of predicting dengue outbreaks up to 3 months in advance based on ensemble models [8]. The system incorporated meteorological, sociodemographic, epidemiological, and preventive data to predict dengue outbreaks, achieving an overall accuracy of 69% [8]. From September to December 2025, E-Dengue was piloted to guide proactive source reduction in An Giang and Tay Ninh [25]. In this pilot, we applied the National source reduction procedure in which households were visited by HVs to provide health education and encourage larval source reduction practices for dengue prevention. A key feature was that this routine source reduction process was informed and triggered by the E-Dengue alerts, rather than the exceedance of observed dengue cases over the risk thresholds, as in routine operation. Dengue outbreak alerts were generated by the E-Dengue system and communicated to CDCs by the study team in the National Institute of Hygiene and Epidemiology (NIHE). The pilot was administratively managed by Departments of Health (DHs), with technical guidance and monitoring provided by CDCs. Based on EWS alerts, the CDCs coordinated with RHCs to develop an implementation plan and operate the source reduction. The RHCs subsequently worked with CPCs and CHSs to mobilize HVs and engaged other relevant stakeholders. Source reduction activities were then delivered at the household level through HV household visits under the coordination of CHSs and supervision of RHCs and CDCs [25].

### Study design

A qualitative study was conducted to explore the barriers to the operation of EWS-informed proactive source reduction [26,27]. This approach was selected to enable in-depth exploration of end-users’ perceptions and experiences in implementing proactive source reduction informed by EWS [28,29]. The conceptual framework presented in Fig 1 was developed by adapting frameworks from previous studies in Vietnam and Malaysia that examined barriers and community needs in implementing source reduction for dengue prevention [30,31]. This framework informed the analytical focus of this study on barriers related to the implementation of EWS-informed source reduction.

**Fig 1:**
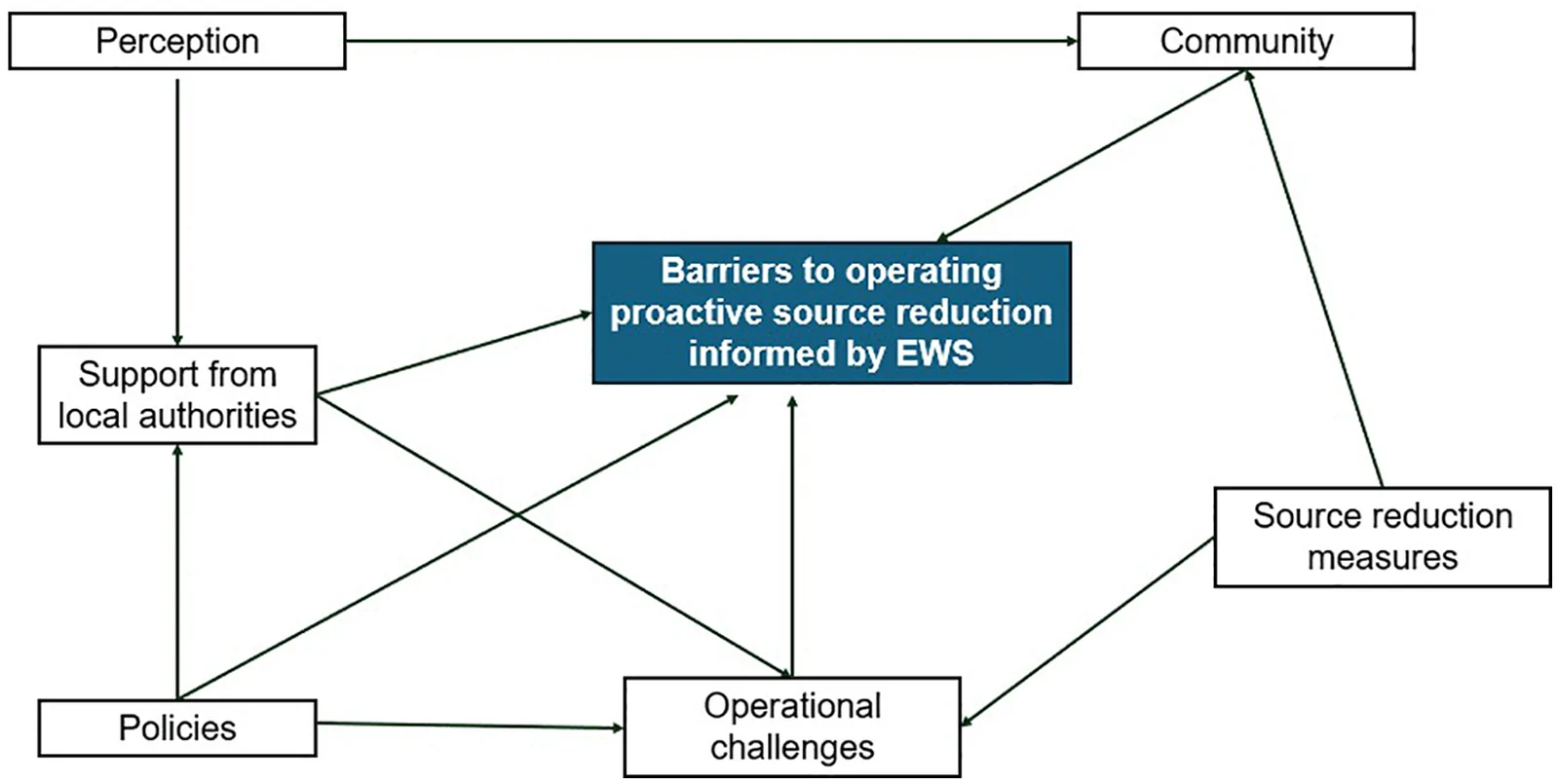
Conceptual framework of barriers to operating EWS-informed proactive source reduction.

Study participants were end-users involved in the implementation of the EWS-informed proactive source reduction during the E-Dengue pilot [26], representing multiple levels of the health system, local authorities, and communities, including:

- DHs: all staff who were responsible for medical professional administration and involved in the E-Dengue pilot, including 1 in An Giang and 1 in Tay Ninh province.
- CDCs: all staff who were responsible for dengue control and involved in the E-Dengue pilot, including 1 in An Giang and 1 in Tay Ninh province.
- RHCs: all staff who were responsible for communicable disease control and involved in the E-dengue pilot, including 1 in An Giang and 1 in Tay Ninh province.
- CPCs: all leaders who mobilised resources for the E-Dengue pilot, including 1 in An Giang and 1 in Tay Ninh province.
- CHSs: staff who were responsible for the field implementation of dengue control and involved in the E-Dengue pilot, including 2 in An Giang and 2 in Tay Ninh province.
- HVs involved in the E-dengue pilot and representing communities in their assigned areas, including 2 in An Giang and 1 in Tay Ninh province.

Participants were purposively recruited from all administrative and operational agencies across provincial, district, commune, and community levels involved in the EWS-informed source reduction. As health agencies and CPCs typically assigned one to two personnel to oversee the management and implementation of the E-Dengue pilot, all technical health staff from DHs, CDCs, RHCs, and CPC leaders involved in the pilot were invited to participate in the interviews. Staff of CHSs and HVs who represented communities and served as the operational workforce for source reduction were purposively recruited from communities implementing EWS-informed source reduction. The recruitment of CHS staff and HVs continued until data saturation was achieved, defined as the point at which no new information emerged from subsequent interviews [32]. To assess data saturation among HVs, information from each interview was summarised in a checklist to monitor the emergence of new information (S1 Checklist).

### Data collection

In-depth interviews were conducted from September to October 2025 in provinces where the pilot of E-Dengue was implemented [25]. These interviews were conducted using a semi-structured interview (S1 Text) designed to explore barriers experienced by local end-users in implementing EWS-informed proactive source reduction.

The interviews were conducted by multiple and diverse researchers from both academic and practice institutions. To minimize potential bias, the interviewers were not involved in dengue control activities and were not affiliated with the Ministry of Health representative agencies or the E-Dengue management team. Before data collection, all interviewers were briefed on current regulations and standard procedures for dengue control, as well as on the implementation of routine dengue control [22]. The interviewers were also informed about the questionnaires and guided to operate the interviews. Interviews were conducted in Vietnamese and face-to-face at the participants’ workplaces, such as in the meeting rooms or in private settings, to ensure participants’ convenience, privacy, and confidentiality. Prior to each interview, participants were informed about the study objectives, interview procedures, and their rights to refuse participation or withdraw from the study at any time without consequences (S2 Text). Opportunities were provided for participants to discuss the study and ask questions with the interviewers before participation. Interviews were conducted only after written informed consent had been obtained (S2 Text). With participants’ consent, interviews were audio-recorded with concurrent note-taking [26]. To ensure trustworthiness and methodological rigor, interview transcripts were returned to selected study participants for member checking, and feedback was incorporated where appropriate to support the credibility of the findings [26].

### Coding and analysis

The recordings were transcribed verbatim by the interviewers who operated the interviews to ensure the completeness, accuracy, and internal consistency of the data. The transcripts were then reviewed by the principal investigator (PI) for quality control, including verification of content accuracy and contextual coherence, before being available for analysis [33].

Data were coded and analysed by an independent analyst who was not involved in the E-Dengue pilot activities but had substantial experience in qualitative research and in the management of dengue control at the provincial level.

Data were analyzed using thematic analysis following the Braun and Clarke framework [34] employing a hybrid approach to coding. We initially adopted a deductive approach to develop a foundational codebook informed by the predefined conceptual framework (Fig 1). To maintain analytical openness, inductive coding was subsequently incorporated to capture emerging concepts that were not represented in the initial framework [35]. The initial codes were refined during familiarisation with the transcripts [34]. The analyst independently coded all transcripts line-by-line using ATLAS.ti software (version 25.0). Although coding was conducted by a single analyst, coding and theme structures were regularly discussed with the research team to ensure consistency in code interpretation and application across transcripts. Coding discrepancies and ambiguous interpretations were reviewed and resolved, with the codebook updated accordingly throughout the analytical process [33,35]. Themes were iteratively generated by grouping conceptually related codes and were continuously refined through iterative comparison across participant groups and interview transcripts. New themes emerging inductively from the data were incorporated when the conceptual framework were not adequately represented within the initial coding framework. Theme definitions were subsequently revised, consolidated, and conceptually distinct themes were separated to ensure that the final themes accurately reflected the underlying data and maintained analytical consistency. Themes were finalized based on consensus between the PI and the research team [27]. The final themes reflected both deductively derived from the predefined conceptual framework and inductively generated insights emerging from data.

## Results

A total of 15 participants were recruited for in-depth interviews. Data saturation was achieved among participants directly involved in community-level source reduction, occurring by the fourth CHS staff interview and the third HV interview. As shown in Table 1, the sample demonstrated balanced gender representation, with higher educational attainment observed among administrative officers and technical health professionals. Interview duration was broadly comparable across participant groups but tended to increase with years of work experience. Administrative and technical professionals generally participated in longer interviews, whereas health volunteers had shorter interviews (S2 Table).

**Table 1:** Characteristics of interviewees and interview durations.

Stakeholders	Number of interviewees	Age	Gender	Education	Work experience	Interview duration (minutes)
Department of Health	2	47	F	Graduated	23 years	50
		32	M	Graduated	3 years	45
Centre for Disease Control	2	27	F	Graduated	2 years	75
		32	M	Graduated	7 years	55
Regional Health Centre	2	31	M	Graduated	4 years	41
		30	M	Graduated	6 years	45
Commune People’s Committee	2	42	F	Graduated	3 months	47
		50	M	Graduated	4 months	45
Commune Health Station	4	46	F	Graduated	2 years	35
		34	M	Graduated	13 years	35
		37	F	Graduated	13 years	47
		34	M	Graduated	10 years	50
Health volunteer	3	54	F	Under-graduated	17 years	35
		39	M	Graduated	10 years	15
		42	F	Under-graduated	10 months	15

Table 2 presents the relationships between themes, subthemes, and related codes identified through thematic analysis. While the initial codes were guided deductively by the predefined conceptual framework [30,31], additional inductive themes emerged during analysis, particularly relating to the limited perception and interpretation of early warning signals and poorly adaptable intervention measures.

**Table 2:** Hierarchical structure of themes and subthemes identified from thematic analysis.

Main themes	Subthemes	Related codes
1. Inadequate supportive policies	1.1. Limited financial and human resources support for EWS-informed source reduction.	• Lack of operational guidance. • Lack of regulatory framework. • Uncertain evidence demonstrating progression toward outbreaks.
2. Limited support from local authorities	2.1. Limited awareness and low prioritization of dengue prevention.	• Limited awareness of EWS among local authorities. • Local authorities lacked public health or medical backgrounds.
3. Operational barriers	3.1 Limited human resources	• Shortage of personnel; density of households in large implementation areas.
	3.2 Time and workload constraints	• Short implementation period; competing routine responsibilities; limited access to households during working time.
	3.3 Technical limitations	• Lacked professional health training.
4. Provider-reported community perception	4.1. Limited perception with EWS-informed risks	• Unfamiliar with EWS-informed source reduction.
	4.2. Limited community participation and engagement	• Perceived responsibility for larval control is placed on local authorities and health agencies. • Focus on treating only large domestic water storage containers.
5. Poorly adaptable measures	5.1. Household water insecurity limits the feasibility of source reduction measures. 5.2. Perceived negative impacts of interventions on water usability and quality.	• Water scarcity and unreliable water supply. • Preferences for rainwater storage. • Water shortage from draining water. • Larvivorous fish may affect water quality.

### Inadequate supportive policies

Policies and regulatory frameworks are important enablers for overcoming constraints related to human and financial resources in vector control activities, which are typically activated only in response to dengue outbreaks identified through explicit epidemiological evidence, such as predefined epidemic thresholds [36,37]. EWS-informed proactive interventions represented a novel approach that required interpretation of probabilistic uncertainty and remained unfamiliar to local stakeholders within routine dengue prevention activities. In addition, the limited empirical evidence demonstrating the operational effectiveness of EWS-informed interventions reduced confidence among local authorities in initiating multisectoral coordination and mobilizing sufficient financial and human resources for proactive source reduction [21]. Consequently, in the absence of supportive policies and regulatory frameworks, local authorities were reluctant to allocate resources for EWS-informed source reduction activities. Participants consistently described decision-making as highly compliance-driven, requiring formal policies, regulatory frameworks, or direction from higher administrative levels. Without explicit policy, guidance from the Ministry of Health, local authorities were reluctant to initiate proactive source reduction informed by EWS activities. As a doctor from DH noted:

*“Everything has to be regulated, everything has to be directed by the Ministry of Health. If the Ministry of Health does not give directions, we don’t dare to come up with something ourselves and do it”.* (DH1)

Similarly, another participant emphasised the need for explicit evidence and official guidance to legitimise action:

*“It’s difficult, very difficult. Even if we go out one or two months earlier, the most important thing is still the People’s Committee. The People’s Committee needs to see explicit evidence, like a standard curve, epidemic threshold, or something like that”.* (CDC2)

In the absence of supportive policies or regulatory frameworks that formally recognised EWS outputs as legitimate triggers for action, EWS-informed proactive source reduction faced substantial constraints in resource mobilisation and budget allocation.

### Lack of local authority support

Community-based dengue prevention requires substantial financial and human resources to achieve sufficient coverage and generate meaningful impacts on dengue control [36]. In addition to supportive policies and regulatory frameworks, effective implementation also depends on the engagement and support from local authorities to coordinate activities and mobilize resources from multisectoral stakeholders. However, such engagement and support were strongly influenced by local authorities’ perceptions of EWS-informed proactive source reduction. Our findings indicated that many local authorities lacked public health or medical backgrounds and were unfamiliar with the probabilistic uncertainty underlying EWS predictions. These factors constrained their engagement and support for proactive source reduction. As a participant explained:

*“Because the People’s Committee staff mostly doesn’t have the medical expertise to advise their leaders in implementing proactive source reduction measures”.* (RHC1)

Even when early warning signals indicated elevated outbreak risk, local authorities were hesitant to allocate financial resources or mobilise multisectoral support because of limited understanding of risk concepts. Without administrative endorsement and budget allocation, frontline health staff faced substantial operational challenges. A participant from RHC noted:

*“For the locations where an outbreak is predicted, without financial support, the local health staff face substantial challenges in operating source reduction activities”.* (RHC2).

Participants emphasised that limited public health expertise among local leaders, combined with existing governance constraints and the lack of a supportive regulatory framework, reduced the translation of predictive risk signals into preventive action. Consequently, proactive source reduction informed by the EWS remained difficult to operationalise.

### Operating source reduction at the community level

Effective source reduction requires intensive efforts to achieve comprehensive household coverage and adequate elimination of mosquito breeding sites [38,39]. Because these activities rely largely on voluntary participation, the quality and consistency of implementation are strongly influenced by the ecological characteristics of the intervention areas as well as the socioeconomic conditions of the workforce involved in source reduction. Our results highlighted those large geographic areas, and high household density substantially limited the feasibility of completing activities within the designated timeframe. These operational constraints were reported across all administrative levels involved in operational source reduction in the community.

*“In large and densely populated areas, the limited three-day timeframe makes it challenging to complete household visits across the entire community.”* (HV1)

*“When visiting around 50 households, even working from morning to afternoon is not enough to complete the task.”* (CHS3)

Beyond geographic scale, limited human resources further constrained implementation. The source reduction mainly relied on the voluntary, and the workforce was largely mobilised from community members who had competing livelihood responsibilities and limited formal accountability. As one health staff member in the CHS noted:

*“The source reduction workforce is mainly mobilised from the community, with limited responsibility, while these individuals still have their own work and other commitments”.* (CHS3)

Participants also noted that many HVs lacked professional health training, which affected the quality and consistency of intervention delivery:

*“Human resource mobilisation is often reactive, as the workforce mobilised from the community is not specialised in health activities and has competing responsibilities”* (CHS4)

Socio-economic conditions introduced additional implementation challenges. In many communities, households were frequently closed during working hours, limiting access to treat water containers and breeding sites:

*“A major challenge is that many houses are closed, as residents go for work. Consequently, water containers are rarely treated.”* (HV2)

Collectively, these findings indicated that even when early warning information was available, the demands of comprehensive community coverage, limited trained personnel, and household accessibility constrained the implementation of effective proactive source reduction.

### Community perceptions

Although the willingness and commitment of households to consistently perform breeding source reduction are key determinants of effective dengue control [40], the low perceived risk of dengue outbreaks informed by the EWS emerged as a critical barrier to implementing proactive source reduction. A key challenge was the mismatch between proactive source reduction informed by EWS and communities’ long-standing experiential knowledge of dengue seasonality [10,20]. As one CHS staff member explained:

*“This approach [proactive source reduction informed by EWS] may be even more challenging, as source reduction conducted during the rainy season is aligned with communities’ long-standing knowledge”.* (CHS1)

In many dengue endemic settings, dengue prevention activities are socially and cognitively anchored to visible seasonal cues, particularly the rainy season. When EWS signals indicate elevated risk outside these familiar periods, communities may question the legitimacy or urgency of the intervention, thereby reducing engagement.

Low risk perception and complacency further limited proactive household participation. As noted by an RHC doctor:

*“Some people are still quite complacent. Even when there is a dengue case in the household, especially involving a child, they do not pay enough attention. Water containers remain around the house, including unused ones, and mosquito larvae are still present.”* (DH2).

This reflected a gap between knowledge of dengue and consistent preventive practice compliance, even in high-risk contexts.

Participants also emphasised that many community members perceived dengue prevention, particularly the source reduction, as the responsibility of local authorities and health staff rather than households. A CPC leader remarked:

*“There are still some people in the community who don’t really care about the source reduction for dengue prevention. They think this work is the responsibility of the local authorities and local health staff. Because of that, it affects dengue control.”* (CPC1).

Such externalisation of responsibility undermined the required household engagement for effective source reduction, especially when EWS-informed source reduction depends on timely and widespread household participation.

Additionally, the diversity of small water-holding containers presented practical challenges to sustained household-level control. Participants highlighted that households were focused on treating only large domestic water storage containers but overlooked items, such as coconut husks, discarded objects, ornamental pots, and flowerpots, which frequently serve as persistent breeding sites:

*“Households often overlook small miscellaneous items, such as coconut husking, and discarded things. Therefore, they do not eliminate them during source reduction.”* (CDC2).

*“With things like ornamental pots and flowerpots, people don’t really pay attention. Water stays in them in the rainy season, and there are mosquito larvae.”* (HV2).

Unfamiliarity with EWS-informed interventions, reliance on authorities, low perceived personal responsibility, and practical challenges in eliminating diverse breeding sites collectively impeded proactive household-level source reduction. These findings suggested that successful operationalisation of EWS-informed dengue control requires not only institutional and policy support but also a shift in community risk perception and ownership of preventive action.

### Poorly adaptable intervention measures

Poorly adaptable intervention measures emerged as a significant barrier to implementing proactive source reduction at the household level. In the Mekong Delta Region (MDR), concerns regarding water scarcity and unreliable water supply, together with preferences for rainwater based on perceptions of lower cost, better water quality, and greater supply security [24], collectively encouraged households to store water in multiple jars and containers for daily use. Consequently, some source reduction measures, such as draining stored water or introducing larvivorous fish, were perceived as impractical or unacceptable.

*“Water containers are abundant, but due to water scarcity, it is not feasible to empty them all. Interventions such as emptying or treating water with larvivorous fish leave households without usable water, which can lead to resistance from the community.”* (RHC1)

Draining water containers directly threatens household water security, while adding larvivorous fish may affect water quality or usability for cooking and drinking. Consequently, these measures could generate resistance rather than cooperation, particularly when implemented proactively based on EWS signals rather than visible outbreaks.

These constraints were further intensified in areas affected by extreme climate-related events, including drought and saltwater intrusion. As noted by a CHS staff member:

*“Because water is limited during drought or salinization, residents maintain multiple water containers. In the rainy season, they still rely on a main water storage container, making it impractical to empty it even when mosquito larvae are present.”* (CHS2)

Even during the rainy season, reliance on stored water persisted due to unstable supply systems. Under such circumstances, expecting households to empty or release fish into their water storage practices is unrealistic.

Overall, intervention measures that failed to account for local ecological and socio-economic conditions, particularly in water scarcity areas, posed a critical barrier to community engagement. These findings suggested that EWS-informed proactive source reduction must be context-adapted, balancing larval control objectives with household water security to ensure feasibility and acceptability.

## Discussion

This study provided empirical insights into how multiple levels of the local health system and community interpreted and operationalised EWS-informed proactive dengue interventions, while further refining an implementation-oriented conceptual framework grounded in real-world implementation experiences. The findings indicated that barriers related to weak policy support, limited authority engagement constrained implementation, operational challenges hindered field execution, and prevailing community perceptions undermined effective household-level source reduction.

Despite numerous EWSs that have been developed and proven to have high predictive performance [8,20], rigorous evidence on their effectiveness and cost-effectiveness remains limited. This evidence gap constrains policy development and the establishment of regulatory frameworks to support the use of EWS in dengue control [21,41]. In the absence of explicit policy and legal frameworks, the engagement of local authorities and relevant stakeholders in supporting EWS-informed source reduction remained limited. Similar barriers have been documented in previous studies in Vietnam and Malaysia, where dengue control efforts were constrained by unclear mandates and insufficient regulatory frameworks [30,42]. These findings underscore the need for a systematic and long-term approach to EWS development that generates policy-relevant evidence and facilitates integration into routine dengue surveillance and control systems.

Proactive source reduction informed by EWS represented a relatively new approach to dengue control, requiring an understanding of probabilistic risk and early warning signals [43,44]. Local authorities often lack specific public health expertise, and decision-making is typically guided by concrete indicators such as outbreak thresholds [22,44]. Given limited resources, priority was frequently allocated to outbreak response and other emergency events. As a result, support for EWS-informed proactive activities remained limited, constraining field implementation. Although WHO guidance and recent studies emphasise that effective dengue vector control extends beyond the health sector and requires coordinated engagement of local authorities and multiple stakeholders [31,45,46], inadequate commitment at the local level may undermine proactive source reduction, particularly in geographically large areas with hard-to-reach households and risk sites, including unclaimed land, inaccessible households, and diverse risk water containers. These findings suggested that strengthening risk communication and improving understanding of EWS among decision-makers may facilitate greater engagement and enhance the operationalisation of proactive source reduction informed by EWS.

Similar to conventional source reduction activities, persistent community perceptions continued to constrain the implementation of EWS-informed proactive source reduction for dengue control [47]. Our findings suggested that mobilising households to undertake source reduction in response to EWS alerts, particularly during periods of low transmission, remained challenging. This reflected a misalignment between EWS-informed proactive approaches and entrenched community perceptions of dengue as a predominantly seasonal disease associated with the rainy season [48,49]. Consequently, interventions triggered outside peak transmission periods may be perceived as unnecessary, reducing engagement and compliance with intervention measures. These findings highlighted the importance of adapting community risk communication strategies to better align with forecasting concepts and, EWS-informed dengue control efforts.

Community acceptance and adherence are central to effective dengue prevention. Evidence from Cuba demonstrated that sustained community participation could significantly reduce larval positive containers [50], while studies in Vietnam and Malaysia have identified limited community engagement as a persistent barrier to vector control effectiveness [30,31]. Building on this literature, our findings provide deeper insight into the drivers of limited acceptance and adherence in the context of EWS-informed proactive source reduction. Similar to observations in other dengue-endemic settings, households commonly viewed source reduction as the responsibility of local authorities and health staff [51]. This reliance may weaken community ownership and impede the sustainability of larval control efforts, as previously documented in Malaysia [31]. In addition, the inflexible implementation of source reduction measures, without adequate adaptation to local environmental and socioeconomic conditions, further constrained community acceptance. For example, recommendations such as water disposal or releasing fish into water containers were perceived as impractical in places and periods of water scarcity.

Our findings highlighted a broader governance challenge in dengue control, where routine responses remain largely reactive and outbreak-driven. Integrating EWS into routine practice represented a necessary shift towards risk governance [43], in which preventive action is triggered by risk signals rather than observed case surges. However, implementation of this approach was shaped by interactions across multiple system levels. At the governance level, the absence of supportive policies and operational mechanisms limited the ability of local authorities to mobilize multisectoral coordination and resources for proactive interventions. At the operational level, health professionals experienced difficulties translating probabilistic early warning signals into actionable vector control activities within existing outbreak-response systems. At the community level, EWS-informed proactive actions often conflicted with prevailing perceptions that dengue prevention should occur mainly during the dengue season or following visible outbreaks. These differing perspectives across health sectors, local authorities, and communities reduced shared understanding of outbreak risks and constrained support for proactive interventions. Together, these findings suggested that implementation of EWS-informed dengue prevention was shaped not only by prediction accuracy but also by the interactions among policies, governance structures, operational practices, and risk perceptions across multiple levels.

This study was guided by a predefined conceptual framework addressing barriers in dengue control, enhancing the methodological coherence and comparability of findings with previous research [30,31,43]. To our knowledge, this is among the first to apply a predefined framework to examine barriers to EWS-informed proactive source reduction. While previous studies on dengue prevention have primarily focused on operational constraints in vector control activities driven by information from passive surveillance systems, such as limited resources and weak community participation [30,36], our findings further contributed to the literature by demonstrating that implementation of EWS-informed proactive source reduction involved additional barriers operating across multiple levels from the health system to local administrative agencies and the community. Collectively, the findings advanced conceptual understanding of governance and behavioural barriers affecting implementation of EWS-informed proactive dengue prevention.

Several limitations should be acknowledged. First, the relatively small sample size may have limited the breadth of perspectives captured and potentially influenced the findings, although this study included all health staff and local authorities involved in the E-Dengue pilot implementation across the two study provinces. However, most health professionals had substantial experience in dengue control, ranging from 2 to 23 years (Table 1), which likely strengthened their ability to identify and describe adequately barriers encountered during routine dengue prevention practices in their local settings. Second, the absence of direct community-level participants may have limited the ability of this study to fully capture community perceptions regarding EWS-informed proactive source reduction. These perspectives may have been partially represented through CHS staff and HVs, who were community members and were nominated to support the implementation of dengue interventions within their assigned communities. Although data saturation for CHS staff and HV interviews was reached after interviewing 4 CHS staff and 3 HVs, most participants had extensive experience in community-based dengue prevention activities (more than 10 years), which likely strengthened their ability to identify and describe community perceptions of dengue prevention practices. However, this limitation highlighted the need for future studies to include broader community representation to strengthen contextual understanding and triangulation of findings. Third, the relatively short pilot implementation period may have limited participants’ exposure to full range of operational challenges related to the proactive source reduction triggered by the EWS. Moreover, because the source reduction is a routine component of dengue control in Vietnam, participants may not have clearly distinguished between routine practices and activities triggered by EWS alerts. Collectively, these may have influenced the interpretation of participants’ experiences. Finally, Vietnamese contextual and sociocultural factors may also influence both implementation barriers and interpretation of the findings, potentially limiting the transferability to broader scales. Therefore, the conceptual framework and findings from this study should be further examined across different sociocultural and implementation settings to assess their broader applicability and generalizability for EWS-informed proactive source reduction.

## Conclusions

This study provides empirical insights into how multiple levels of the local health system interpret and operationalise EWS-informed proactive dengue interventions within routine practice, while further refining an implementation-oriented conceptual framework grounded in real-world implementation experiences. Findings indicated that implementation of EWS-informed proactive source reduction within routine dengue control may require supportive policy and regulatory frameworks, sustained stakeholder and community engagement, and strengthened risk communication through multiple channels, targeting both communities and local authorities to engage and facilitate timely action. Adapting source reduction measures to local environmental and socio-economic contexts may further improve feasibility and acceptability. However, given the context-specific nature of this study, the findings should be interpreted cautiously and may not be directly transferable to other settings with different governance structures, health systems, and sociocultural contexts. Further studies in diverse implementation settings are needed to assess the broader applicability of the proposed conceptual framework for EWS-informed proactive dengue prevention.

## Supporting information

S1 Table Dengue burden and larval index in An Giang and Tay Ninh from 2021 to 2025. (DOCX)

S1 Checklist Checklist for monitoring data saturation. (DOCX)

S1 Text In-depth Interview guide. (DOCX)

S2 Text Information and consent. (DOCX)

S2 Table Key characteristics of study participants. (DOCX)

S3 Text Study plan. (DOCX)
