# Supplementary material for: Barriers in operating proactive source reduction informed by an early warning system for dengue prevention: A qualitative study from end-user perspectives in Vietnam
Source: PLoS Negl Trop Dis. 2026 Sep 25;20(9):e0014754. doi: 10.1371/journal.pntd.0014754 (PMC13649133; doi:10.1371/journal.pntd.0014754)
Supplement: Supplementary file 1 [file pntd.0014754.s007.pdf]

Luis Adrián Díaz, Ph.D.
Academic Editor
PLOS Neglected Tropical Diseases

Dear Dr. Díaz,

Please find attached my review of manuscript **PNTD-D-26-00374**.

This review is structured into three sections: (1) confidential comments to the editor, (2) general comments to the authors, and (3) major comments, comprising 15 subsections that provide detailed recommendations to strengthen the manuscript, from clarifying the study's contribution to the final recommendation.

The review addresses both the content and presentation of the manuscript, with the aim of supporting the authors in improving its clarity, rigor, and overall contribution.

I hope this review is helpful in informing your editorial decision.

Sincerely,

c1e18be7-f604-449a-9474-1046deceff17

Firmado digitalmente por c1e18be7-f604-449a-9474-1046deceff17
Fecha: 2026.03.24 21:30:57 -06'00'

Benicio Gutiérrez-Doña, Ph.D.
Research Professor of Psychology
Psychological and Behavioral Sciences Research Program
Universidad Estatal a Distancia (UNED), San José, Costa Rica

Reviewer report manuscript PNTD-D-26-00374, PLOS Neglected Tropical Diseases

1. Confidential comments to the editor

The manuscript *"Barriers in operating proactive source reduction informed by an early warning system for dengue prevention: A qualitative study from end-user perspectives in Vietnam"* addresses an important and operationally relevant topic: barriers to implementing early warning system (EWS)-informed proactive source reduction for dengue control in Vietnam.

The topic is clearly within the scope of PLOS Neglected Tropical Diseases and aligns well with the journal's emphasis on implementation challenges in endemic settings.

The study is methodologically appropriate and provides useful multi-level insights from Vietnamese stakeholders involved in dengue prevention. The qualitative design is sound, and the manuscript is generally well written and clearly structured.

In my view, this is a publishable study with clear practical relevance, but it would benefit from strengthening its conceptual framing, analytical depth, and reporting clarity to fully meet Q1 standards.

However, several issues related to the theoretical framework, methodological approach, data and discussion require attention to improve the manuscript.

I recommend major revision.

2. General comments to the authors

This manuscript presents a timely and relevant qualitative investigation of barriers to implementing EWS-informed proactive source reduction for dengue control. The focus on translating surveillance systems into actionable vector control is highly valuable, particularly in endemic contexts where operational challenges often limit the effectiveness of prevention strategies.

The study is well designed, and the inclusion of multiple stakeholder perspectives across different levels of the health system is a clear strength. The manuscript is also clearly written, logically structured, and accessible to a broad readership.

Importantly, the findings provide useful insights into multi-level barriers, including governance, operational constraints, and community-level dynamics. These insights have practical implications for improving dengue control programs in Vietnam.

To further strengthen the manuscript and enhance its contribution to the field, I encourage the authors to deepen the analytical interpretation of their findings, more clearly articulate the study's conceptual contribution, and improve methodological transparency in selected areas.

In addition, expanding the conceptual scope to better reflect the WHO multi-component nature of dengue prevention and clarifying the role of community-level perspectives would further enhance the manuscript's relevance and impact.

Overall, this is a valuable and promising study, and I believe it can make a meaningful contribution to the literature following revision.

3. Major comments

1. Clarifying and strengthening the study's contribution

The manuscript addresses an important and relevant topic; however, the distinction between this study and the existing literature on dengue control barriers could be more

explicitly articulated. In its current form, the contribution appears somewhat implicit rather than clearly positioned within the broader field.

To strengthen the manuscript, it would be helpful for the authors to more clearly define what is specifically novel about focusing on EWS-informed implementation, how the present study adds to or extends existing knowledge (literature) on implementation barriers, and whether the findings can be situated within a broader conceptual or operational framework.

The authors should explicitly clarify what is novel in this study and how it differs from previous research on EWS implementation. This implies positioning the study within existing literature, stating the study´s contribution clearly, linking the findings to a conceptual or operational framework, making the discussion more analytical and less descriptive.

Clarifying these elements throughout the manuscript would strengthen its theoretical, methodological, and practical contributions, and improve its positioning within the international literature.

2. Enhancing analytical depth of results

The results are clearly presented and well structured, offering a coherent thematic overview of barriers across multiple system levels. However, the analysis tends to remain largely descriptive, focusing on identifying and illustrating barriers rather than fully exploring their underlying dynamics.

The manuscript would benefit from a deeper analytical treatment that examines why these barriers arise, how they interact across institutional and community levels, and whether there are meaningful variations or divergences in perspectives among stakeholder groups.

For example, triangulation with a broader sample (e.g., non-participating community members) would enhance the interpretive depth of the findings and support a more comprehensive understanding of information dynamics across sources and levels.

Strengthening this analytical dimension would significantly enhance the interpretive depth and scientific value of the findings.

3. Strengthening conceptual framing in the discussion

The discussion provides a useful synthesis of the findings but could more strongly elevate them to a conceptual level. At present, the interpretation largely confirms existing knowledge rather than advancing broader understanding.

The manuscript would benefit from more explicit engagement with implementation science or health systems frameworks, allowing the findings to be interpreted within a more structured theoretical context.

In particular, articulating a clear conceptual takeaway, such as a model of multi-level barriers or implementation dynamics, would enhance the manuscript's contribution and relevance for an international audience. It would be helpful for the authors to clarify whether they are open to identifying new categories emerging from the data and, if so, how these insights could inform refinements to their initial analytical framework.

That is, adopting a more iterative inductive–deductive approach to the analysis, allowing for the identification of keywords, key phrases, and emergent codes and categories, which can then be organized into a relational thematic structure aligned with the pre-defined logic model.

4. Calibrating policy and practice implications

The manuscript offers valuable practical insights that are directly relevant to dengue (vector) control programs. In some instances, however, the strength of the policy recommendations may slightly exceed the empirical scope of the qualitative data and the specific context from which data were gathered.

While the recommendations are plausible and useful, they would benefit from being more explicitly framed as evidence-informed interpretations grounded in stakeholder perspectives within a culturally embedded context. Barriers to proactive EWS implementation may also stem from sociocultural determinants not accounted for in the current analytical framework.

Vietnamese culture may play a significant role in shaping contextual barriers to proactive EWS implementation, including seasonal risk perceptions, tensions between official and experiential knowledge, collective versus individual responsibility, livelihood priorities, water storage practices, misalignment between official communication and local norms, and social norms that may limit behavioral change. Does the current research framework adequately consider the Vietnamese cultural context?

This adjustment would ensure that the conclusions remain appropriately aligned with the methodological approach while preserving their practical value for Vietnam.

5. Clarifying sampling and saturation

The inclusion of stakeholders across multiple levels of the health system represents a clear strength of the study. Nevertheless, two issues warrant further attention: (i) the qualitative representativeness of the sample (i.e., its transferability potential), and (ii) the transparency of the sampling procedures.

In particular, the study would benefit from expanding the number of key informants to include not only formal authorities but also informal community leaders and their social networks.

In addition, the manuscript would benefit from clarifying how participants were identified and recruited, indicating (where feasible) the total number of eligible individuals within each stakeholder category, and explaining how data saturation was assessed across the dataset.

Providing this information would enable readers to better evaluate the robustness and coverage of the qualitative sample.

6. Expanding transparency of the analytical process

The description of the thematic analysis is appropriate and indicates the use of established qualitative methods. However, greater detail regarding the analytical process would strengthen the manuscript.

Specifically, it would be helpful to clarify whether coding involved more than one researcher, how coding consistency was ensured, and how themes were developed or emerged from initial codes.

Additionally, further elaboration on the balance between framework-driven (deductive) and emergent (inductive) analysis would enhance transparency and allow readers to better assess the rigor of the analytical approach.

Benicio Gutiérrez-Doña, Ph.D. Research Professor of Psychology. Email: beniciogd@uned.ac.cr

7. Reflexivity and researcher positioning

The manuscript would benefit from the inclusion of a reflexivity statements. While the study appropriately describes aspects of the data collection process, it provides limited reflection on how the researchers' backgrounds, perspectives, or prior involvement in dengue-related work may have influenced data collection and interpretation. Including a discussion of researcher positioning and any steps taken to minimize potential bias would further strengthen the methodological rigor of the study.

For example, did the research team include members with experience in public health or dengue prevention that may have influenced data interpretation?

Were steps taken to minimize potential bias, such as using interviewers not involved in the intervention or employing collaborative coding?

How did the team ensure that findings remained grounded in participants' perspectives (e.g., through reflexive discussions or iterative analysis)?

Finally, how did the authors' epistemological stance (e.g., positivism/post-positivism, interpretivism/constructivism, critical or hermeneutic approaches, pragmatism, phenomenology, grounded theory, or critical realism) shape data collection, interpretation, and claims about knowledge?

8. Data availability statement (important for PLOS compliance)

The current data availability statement indicates that *data are available upon reasonable request*, which may not fully align with PLOS data sharing policies.

To ensure compliance, the authors are encouraged to either provide access to the data through an appropriate public repository or clearly justify any restrictions (e.g., legality) related to confidentiality or ethical considerations.

In the latter case, it would be important to describe a formal and transparent process through which qualified researchers may request access to the data.

Some countries restrict data sharing due to legal regulations. What is the case in Vietnam?

9. Clarification of informed consent

The manuscript would benefit from a more explicit description of informed consent procedures. In particular, it should be clarified whether consent was obtained in written or oral form and justify and justify this choice. If oral consent was used, the authors should describe how it was documented and how this approach was approved by the relevant ethics committees. This clarification is important for ensuring transparency and alignment with PLOS-NTD journal requirements.

10. Transferability of findings

While the study is situated within a specific national and programmatic context, its findings have potential relevance for other dengue-endemic settings. The manuscript would benefit from a more explicit discussion of transferability, including a clear distinction between context-specific findings and those that may be applicable more broadly. Expanding this discussion would enhance the manuscript's international relevance and contribution.

For example, to what extent do the authors consider the identified barriers to be specific to the Vietnamese provincial context versus applicable to other dengue-endemic settings? Which findings are most likely transferable, and under what conditions or health

system characteristics? How might contextual factors (e.g., governance, resources, community engagement, and cultural determinants) influence the applicability of these findings elsewhere? Finally, is the sample qualitatively representative enough to capture informal levels of health prevention?

11. Scope of preventive strategies considered

The manuscript provides a focused and valuable analysis of barriers to proactive source reduction through EWS implementation. However, a substantial body of literature indicates that dengue prevention is inherently multi-dimensional. Global strategies, including those recommended by the World Health Organization, encompass not only vector control but also individual-level protective behaviors, such as the use of mosquito repellents, protective clothing, household barriers, and fumigation.

In its current form, the study primarily conceptualizes prevention in terms of vector life-cycle interruption, without fully considering how human protective behaviors may complement or interact with these strategies.

To enhance conceptual completeness, the authors could adopt a broader view of dengue prevention as a multi-component system, clearly delineating the boundaries of their study while discussing how individual-level behaviors may interact with, or compensate for, limitations in vector control. Integrating this perspective would strengthen the manuscript's alignment with global public health frameworks.

For example, how do the authors conceptualize the role of individual-level protective behaviors (e.g., repellents, protective clothing, household barriers) in relation to EWS-informed source reduction? Could these be framed as complementary components within a broader, multi-dimensional prevention framework?

12. Sample composition and opportunities for enhanced triangulation

The inclusion of multi-level institutional stakeholders is a notable strength; however, the sample does not include members of the general population in lower versus higher-risk communities, nor does it explicitly capture variations across key demographics or health-related characteristics (e.g., age, sex, prior dengue infection). This may limit insight into lived experiences of dengue prevention and reduce opportunities to triangulate institutional official perspectives with community realities.

The manuscript could be strengthened by incorporating more community participants in the sample, greater demographic variation, and potentially focus group methodologies to enhance triangulation and interpretive depth.

In particular, how might the absence of representative community-level participants, especially individuals from higher- versus lower-risk populations, have influenced the findings? How might the inclusion of such perspectives (e.g., by age, sex, or prior dengue infection) enhance triangulation and the interpretation of implementation barriers?

13. Need a comprehensive review of references

Issues found with Vancouver format references style (examples).

1. Dengue [Internet]. [cited 2026 Jan 18]. Available from: https://www.who.int/newsroom/fact-sheets/detail/dengue-and-severe-dengue

Issues: The cited webpage is no longer accessible, no author/organization clearly formatted, no year of publication, no publisher location.

2. Dengue - Global situation [Internet]. [cited 2026 Jan 18]. Available from: https://www.who.int/emergencies/disease-outbreak-news/item/2024-DON518
   Issues: Missing organization name at start, missing publisher location (Geneva), missing publication year
24. thuvienphapluat.vn. Law library [Internet]. 2025 [cited 2026 Jan 18]. Decision 3711/QĐ-BYT 2014 National guideline for dengue surveillance and prevention. Available from: https://thuvienphapluat.vn/van-ban/The-thao-Y-te/Quyet-dinh-3711-QD-BYT-2014-huong-dan-giam-sat-phong-chong-benh-Sot-xuat-huyet-Dengue-251556.aspx
   Issues: Website used as author (incorrect format), missing institutional author, missing publisher location.
25. Electronic communicable disease surveillance portal [Internet]. [cited 2026 Jan 18]. Available from: https://gs.vadp.gov.vn/Account/Login
   Issues: No author institution, no publisher/location, no year.

Several web-based references lack essential publication details required by Vancouver style (e.g., author/organization, publisher, location, and year). These should be completed to ensure proper citation and reproducibility.

Other issues examples found with Vancouver format style:

1. **Inconsistent use of journal abbreviations.**
   Some journals are abbreviated (correct Vancouver), others are written in full (incorrect), for example: Journal of Chemical Health Risks, International Journal of Infectious Diseases, PLOS Neglected Tropical Diseases, etc.
2. **The formatting of DOIs** is inconsistent across references (e.g., use of "doi:", inclusion of URLs, and additional identifiers such as PubMed IDs). A uniform Vancouver style should be applied, using "doi:xxxxx" for journal articles and avoiding redundant elements.

Additional inconsistencies are also present.

14. Language/syntactical issues examples are shown within […]

1. Missing verb? "At the community level, the perception that dengue risks **[occur]** only during the rainy season,"
2. Incomplete clause? "The technical oversight **[is provided]** by provincial Centers for Disease Control (CDC) and Regional Health Centres (RHC)."
3. Missing verb? "The community-based implementation **[is]** led **[by]** Commune Health Stations (CHSs)…"
4. Sentence fragments: "Village-wide expansion **[is implemented]** when outbreak thresholds are exceeded, ~~or~~ **[when]** more than three outbreaks reported within the same village in 2 consecutive weeks, or **[when]** incidence in 2 consecutive weeks **[is]** greater than 150 cases/100,000 persons
5. Incorrect wording? The study collected data from interviews; no ~~identical~~ **[identifiable]** information was recorded.

6. Phrasing issue? "As **[shown]** in Table 1,"
7. Typographical error: "…or treating water with ~~lavivorous~~ **[larvivorous]** fish leave households without usable water,"
8. Missing article? "In the absence of explicit policy and legal frameworks, **[the]** engagement of local authorities…"
9. Data availability inconsistency? Page 4: "Data may be made available upon reasonable request and with approval…", Page 18: "Data are available upon reasonable request…" It's better to be standardized.

The manuscript contains grammatical, syntactical, and wording inconsistencies (e.g., missing verbs, incomplete clauses, and typographical errors). Although these do not affect the scientific content, they reduce clarity and readability. A careful line-by-line language revision is recommended to ensure accuracy, consistency, and coherence throughout the manuscript.

15. Final recommendation

I recommend **major revision**. The manuscript requires substantial improvements in clarifying and strengthening the study's contribution, enhancing the analytical depth of the results, and strengthening the conceptual framing in the discussion. Additional revisions are needed to calibrate policy and practice implications; clarify sampling procedures and saturation; increase transparency of the analytical process, including reflexivity and researcher positioning; and clarify the data availability statement (important for PLOS compliance) and informed consent procedures. Further improvements include more explicit consideration of the transferability of findings, adoption of a broader scope of preventive strategies, expansion of the sample to enhance opportunities for triangulation, a comprehensive review of the reference list, and correction of language and syntactical issues. This review provides detailed recommendations addressing each of the points outlined above.
