# Supplementary material for: Barriers in operating proactive source reduction informed by an early warning system for dengue prevention: A qualitative study from end-user perspectives in Vietnam
Source: PLoS Negl Trop Dis. 2026 Sep 25;20(9):e0014754. doi: 10.1371/journal.pntd.0014754 (PMC13649133; doi:10.1371/journal.pntd.0014754)
Supplement: Supplementary file 2 [file pntd.0014754.s001.docx]

**S1 Table.** Dengue burden and larval index in An Giang and Tay Ninh from 2021 to 2025.

| Year | An Giang | |  | Tay Ninh | |  | Total | |  |
| --- | --- | --- | --- | --- | --- | --- | --- | --- | --- |
|  | **Cases** | **Deaths** | **BI** | **Cases** | **Deaths** | **BI** | **Cases** | **Deaths** | **BI** |
| 2021 | 3170 | 0 | 28 | 3485 | 3 | 59 | 6655 | 3 | 44 |
| 2022 | 22196 | 5 | 49 | 27820 | 17 | 34 | 50016 | 22 | 42 |
| 2023 | 7428 | 3 | 52 | 5080 | 6 | 32 | 12508 | 9 | 42 |
| 2024 | 2926 | 2 | 44 | 2988 | 1 | 37 | 5914 | 3 | 41 |
| 2025 | 6164 | 1 | 36 | 14190 | 3 | 30 | 20354 | 4 | 33 |
| Average 2021-2025 | 8377 | 2 | 42 | 17013 | 6 | 39 | 19089 | 8 | 41 |

^a^Breteau Index (BI) is the number of water-holding containers positive for Aedes larvae and/or pupae per 100 houses inspected.
