# Supplementary material for: Barriers in operating proactive source reduction informed by an early warning system for dengue prevention: A qualitative study from end-user perspectives in Vietnam
Source: PLoS Negl Trop Dis. 2026 Sep 25;20(9):e0014754. doi: 10.1371/journal.pntd.0014754 (PMC13649133; doi:10.1371/journal.pntd.0014754)
Supplement: Supplementary file 3 [file pntd.0014754.s002.docx]

**S2 Text.** Checklist for monitoring data saturation.

| **Interview** | **Policy** | **Local authority engagement** | **Operational barriers** | **Community perception** | **New information** | **Data saturation** |
| --- | --- | --- | --- | --- | --- | --- |
|  |  |  |  |  |  |  |
|  |  |  |  |  |  |  |
|  |  |  |  |  |  |  |
|  |  |  |  |  |  |  |
|  |  |  |  |  |  |  |
|  |  |  |  |  |  |  |
|  |  |  |  |  |  |  |
|  |  |  |  |  |  |  |
|  |  |  |  |  |  |  |
|  |  |  |  |  |  |  |
|  |  |  |  |  |  |  |
|  |  |  |  |  |  |  |
|  |  |  |  |  |  |  |
