# Supplementary material for: Barriers in operating proactive source reduction informed by an early warning system for dengue prevention: A qualitative study from end-user perspectives in Vietnam
Source: PLoS Negl Trop Dis. 2026 Sep 25;20(9):e0014754. doi: 10.1371/journal.pntd.0014754 (PMC13649133; doi:10.1371/journal.pntd.0014754)
Supplement: Supplementary file 4 [file pntd.0014754.s003.docx]

**S3 Text.** In-depth Interview Guide

**Target participants: Healthcare workers**

**Section 1. Interviewee Information**

1. Full name:  Age:
2. Current position, responsibilities, and role?
3. Years and type of professional experience?
4. Functions and responsibilities of your organisation/unit?
5. Your involvement in dengue prevention and control activities at the local level?

**Section 2. Current Situation of Dengue Prevention and Control**

1. What is the current dengue situation in your area?

- How is dengue fever currently occurring in your locality?
- In recent years, during which period has dengue been most severe?
- Which areas in your locality have the most complex or high-risk dengue situation?

2. How are routine source reduction and dengue control activities implemented, both routinely and during outbreaks?

- How are you involved in these activities? What is your role?
- How are these activities carried out step by step in practice?
- How would you assess the effectiveness of these activities?

**Section 3. Challenges in Source Reduction Activities Based on Dengue Early Warning**

1. What difficulties do you encounter when implementing larval source reduction activities? Probe further about specific challenges, for example:

- Workload and task volume
- Support from local authorities, sectors, and the community
- Funding and resource availability
- Technical guidance, training, and skills
- Other challenges?

2. In your opinion, what measures could help address these difficulties?
3. Regarding early warning information:

- How would larvae source reduction activities be implemented differently compared to current practice?
- What potential challenges might arise?

**Target participants: Health volunteers**

**Section 1. Interviewee Information**

1. Full name:  Age:
2. Main occupation?
3. How long have you worked as a health collaborator?

**Section 2. Current Situation of Dengue Prevention and Control and Challenges in Larval Source Reduction Activities**

1. Please describe the dengue prevention and control activities you participate in at the local level.

- How are you involved in these activities? What is your role?
- How are these activities carried out step by step in practice?

2. What are the facilitators and challenges when implementing larval source reduction activities? Probe further about challenges, for example:

- Workload.
- Number of participants involved.
- Support from local authorities.
- Community compliance.
- Funding.
- Equipment and supplies.
- Others.

3. What solutions would you suggest to improve the effectiveness of these interventions?
4. With early warning information, what difficulties might arise in implementing larval source reduction activities? How would this differ from current practice?
