# Supplementary material for: Barriers in operating proactive source reduction informed by an early warning system for dengue prevention: A qualitative study from end-user perspectives in Vietnam
Source: PLoS Negl Trop Dis. 2026 Sep 25;20(9):e0014754. doi: 10.1371/journal.pntd.0014754 (PMC13649133; doi:10.1371/journal.pntd.0014754)
Supplement: Supplementary file 5 [file pntd.0014754.s004.docx]

**S4 Text.** Information and consent

**INFORMATION AND CONSENT**

Thank you for your interest in and willingness to participate in our E-DENGUE study. We would like to invite you to take part in this research. The following information explains the purpose of the study and what your participation would involve if you chose to take part.

Before deciding whether or not to participate, you are encouraged to ask us any questions you may have about the study. You may also discuss your participation with others before making your decision.

If you agree to participate, you will be asked to sign the consent form at the end of this document. By signing the consent form, you confirm that you:

1. Have understood the information provided about this study;
2. Have had the opportunity to ask questions and received satisfactory answers; and
3. Voluntarily agree to participate in the study.

A copy of this Information and Consent will be provided to you.

**1. Introduction**

Dengue is an acute infectious disease caused by a virus transmitted by mosquitoes. It can cause severe flu-like symptoms and, in some cases, progress to severe dengue, which may result in serious complications and death. The incidence of dengue has increased more than 30-fold over the past 50 years. It is estimated that 50–100 million dengue cases occur annually across more than 100 endemic countries, placing nearly half of the world’s population at risk.

In Vietnam, approximately 95,000 dengue cases and 70–100 dengue-related deaths are reported each year. Dengue has become endemic throughout the country, particularly in the Mekong Delta region. Although there is currently no specific treatment for dengue or severe dengue, early detection and timely access to appropriate healthcare can significantly reduce mortality. Effective vector control remains a cornerstone of dengue prevention and control.

Vietnam’s current dengue prevention and control programme relies primarily on vector control measures and community awareness activities. Forecasting models based on climate and weather factors have shown potential for predicting dengue transmission.

The study entitled “A user-friendly digital prediction tool for dengue prevention”, abbreviated as the E-Dengue project, was approved by the Ministry of Health under Decision No. 2437/QĐ-BYT dated 8 June 2023. This five-year project (2023–2027) aims to develop and evaluate a user-friendly early warning tool for dengue prevention and control. The project includes the development of an early warning model, creation of the E-Dengue, evaluation of its effectiveness, and assessment of its cost-effectiveness for dengue prevention and control in the Mekong Delta.

The study is funded by the Welcome Trust through the University of Queensland, Australia, and is implemented by the National Institute of Hygiene and Epidemiology (NIHE) in collaboration with relevant partner organizations.

**2. What will happen?**

If you agree to participate, you will be invited to take part in an in-depth interview (IDI) to share your experiences, concerns, challenges, and needs regarding the early warning system (EWS) informed source reduction.

You will also be asked to provide your perspectives on the acceptability, appropriateness, adaptability, and sustainability of this approach. The information collected will help researchers develop culturally appropriate dengue control strategies that can enhance the EWS-informed proactive source reduction. Each interview will last approximately 60 minutes and will be scheduled at a time convenient for you.

**3. Benefits**

Participation in this study is not expected to provide direct personal benefits. However, your contribution will help researchers develop more appropriate strategies to support dengue prevention and control programmes in the community.

You will have the opportunity to share your professional experiences and expertise. You may also benefit indirectly if you become a future user of the E-Dengue tool within dengue prevention and control programmes in your locality.

To compensate for your time and/or travel expenses, you will receive **VND 318,000** for participating in the interview.

**4. Risks**

The interview poses no physical risks to you. However, participation may require some of your personal time, and certain topics discussed during the interview may make you feel uncomfortable or reluctant to answer.

**5. Audio Recording**

The interview will be audio-recorded to ensure that all information shared during the discussion is accurately captured. No one outside the research team will have access to the audio recordings.

You and the interviewer may use pseudonyms during the interview if preferred.

**6. Confidentiality**

Any personal information collected during this study will be kept strictly confidential. We will not disclose your participation or any information you provided to colleagues or any other parties.

The information you provide will be used solely for research purposes. Audio recordings and interview transcripts will be password-protected and stored securely on computers and in locked cabinets. Only authorized members of the research team will have access to the study data.

**7. Your Rights**

Your participation in this study is entirely voluntary. You may choose not to participate or may withdraw your consent and discontinue participation at any time without providing a reason.

Whatever your decision, you will not experience any disadvantages, and your employment or professional responsibilities will not be affected. You may also decline to answer any question that you do not wish to answer.

**8. Contact Information**

If you have any questions, comments, or concerns regarding this study, please contact:

**Principal Investigators**

(Information of Principal Investigators)

**Ethics Committee**

Scientific Management Office

National Institute of Hygiene and Epidemiology, Vietnam

(contact information)

Do you have any questions for us?

**CONSENT TO PARTICIPATE IN RESEARCH**

I have read and understood the information provided above. I have had the opportunity to ask questions, and all of my questions have been answered satisfactorily. I voluntarily agree to participate in this study.

_____________________	______________________	________________________

Participant's Full Name	Participant's Signature	Date (DD/MM/YYYY)

_____________________	____________________

Witness's Full Name	Witness's Signature

_________________________________	_____________________________________

Name of Person Obtaining Consent	Signature of Person Obtaining Consent
