# Supplementary material for: Barriers in operating proactive source reduction informed by an early warning system for dengue prevention: A qualitative study from end-user perspectives in Vietnam
Source: PLoS Negl Trop Dis. 2026 Sep 25;20(9):e0014754. doi: 10.1371/journal.pntd.0014754 (PMC13649133; doi:10.1371/journal.pntd.0014754)
Supplement: Supplementary file 6 [file pntd.0014754.s005.docx]

**S5 Table.** Key characteristics of study participants.

| **Participant ID** | **Affiliation** | **Age (years)** | **Education level** | **Administrative level** | **Primary responsibility** | **Years of experience** | **Interview duration (min:sec)** |
| --- | --- | --- | --- | --- | --- | --- | --- |
| DH1 | Department of Health Tay Ninh | 47 | Graduated | Province | Professional administration | 23 years | 50 |
| DH2 | Department of Health An Giang | 32 | Graduated | Province | Professional administration | 3 years | 45:00 |
| CDC1 | Centre for Disease Control Tay Ninh | 27 | Graduated | Province | Dengue surveillance and response | 2 years | 75:00 |
| CDC2 | Centre for Disease Control An Giang | 32 | Graduated | Province | Dengue surveillance and response | 7 years | 55:00 |
| RHC1 | Regional Health Centre Can Giuoc | 31 | Graduated | Province | Communicable disease response and prevention | 4 years | 41:07 |
| RHC2 | Regional Health Centre Phu Tan | 30 | Graduated | Province | Communicable disease response and prevention | 6 years | 45:00 |
| CPC1 | Commune People's Committee Khanh Hau | 42 | Graduated | Commune | Social and cultural affairs | 3 months | 46:54 |
| CPC2 | Commune People's Committee Phu Binh | 50 | Graduated | Commune | Social and cultural affairs | 4 months | 45:00 |
| CHS1 | Commune Health Station Khanh Hau | 37 | Graduated | Commune | Communicable disease response and prevention | 13 years | 46:45 |
| CHS2 | Commune Health Station Tan Tap | 34 | Graduated | Commune | Communicable disease response and prevention | 13 years | 35:00 |
| CHS3 | Commune Health Station Phu Binh | 46 | Graduated | Commune | Communicable disease response and prevention | 2 years | 35:00 |
| CHS4 | Commune Health Station Long Dien A | 34 | Graduated | Commune | Communicable disease response and prevention | 10 years | 50:00 |
| HV1 | Health volunteer Tan Tap | 54 | Under-graduated | Community | Dengue prevention and response | 17 years | 35:00 |
| HV2 | Health volunteer Phu Binh | 39 | Graduated | Community | Dengue prevention and response | 10 years | 15:00 |
| HV3 | Health volunteer Long Dien A | 42 | Under-graduated | Community | Dengue prevention and response | 10 months | 15:00 |
