# Supplementary material for: Barriers in operating proactive source reduction informed by an early warning system for dengue prevention: A qualitative study from end-user perspectives in Vietnam
Source: PLoS Negl Trop Dis. 2026 Sep 25;20(9):e0014754. doi: 10.1371/journal.pntd.0014754 (PMC13649133; doi:10.1371/journal.pntd.0014754)
Supplement: Supplementary file 7 [file pntd.0014754.s006.docx]

**S6 Text.** Study plan p*repared by Do Kien Quoc – Version 1.0, 14^th^ September 2025.*

**Rationales**

- Dengue poses a significant threat globally and in Vietnam.
- Vector control is pivotal, and breeding source reduction is a fundamental, adaptable component that can be integrated into all strategies and intervention measures. Current vector control is not proactive and relies on the detection of index cases.
- Early Warning System (EWS) provides promising results, but none have been used in the routine practice of dengue prevention and control.
- Evidence on the barriers to proactive source reduction informed by EWS is scarce, limiting the application of this approach in routine practice.

**Research questions**

- What are the barriers to proactive source reduction informed by EWS in dengue control in Vietnam?

**Objective**

- To determine the barriers to proactive source reduction informed by EWS from end-user perspectives in Vietnam.

**Conceptual framework****Figure 1.** Conceptual framework for assessing challenges in breeding source reduction

The barriers in implementing proactive source reduction informed by EWS will be identified from the multifaceted interactions of local authorities, community members, and health staff. Policies, perceptions, and personal experiences shape community engagement, while support from local authorities and technical barriers act as key components influencing the overall implementation.

**Methodology**

This study will be conducted in An Giang and Tay Ninh provinces in the South of Vietnam during the pilot phase of the E-Dengue project. This project will be implemented in the Mekong Delta Region in Vietnam to test the effectiveness of proactive source reduction informed by E-Dengue, an EWS.

We will conduct a qualitative study with a descriptive design to explore the barriers to proactive breeding source reduction informed by EWS. Study participants will be purposively recruited into this study, including participants from the department of health, Centers for Disease Control (CDC), Regional Health Centers (RHC), commune authorities, commune health staff, and health volunteers. We will recruit all available experts in the field of dengue prevention and control. At the commune level, the sample size will follow the principle of data saturation, which means the recruitment will cease once no new information is obtained from additional participants in that category. Within this study, we will conduct in-depth interviews guided by a semi-structured questionnaire to collect data. This approach enables us to seek collective perspectives and descriptions on the challenges in operating and practising breeding source reduction.

Thematic analysis following the framework of Braun and Clark (2006) will be performed on the collected data to identify the barriers to the proactive source reduction as perceived by local authorities, health staff, and the community. ATLAS.ti (version 25.0).

Member checking, multiple and diverse interviewers from academic and practical institutions, independent coder who are not involved in E-Dengue project and representative of Ministry of Health institutions will carry out the interviews and coding process to ensure the methodological rigor for this study.

**Implementation plan**

*Milestones*

This study will be incorporated into the pilot phase of the E-Dengue project from September to November in An Giang and Long An, and aims to achieve the following milestones:

- Complete documents of in-depth interview guidance, semi-structured questionnaire, ICF-consent form, and form for monitoring data saturation in in-depth interview by the first week of October 2025.
- Complete data collection by the end of November 2025.
- Complete data analysis, including thematic analysis, by the end of December 2025.
- Present preliminary findings at the annual project meeting in December 2025.
- Develop first draft of manuscript by the end of January 2026.
- Revise and proceed with the submission by the end of February 2026.

*Delegated tasks*

- Prof. Dung Phung oversees technical consultation, mentorship, and supervision.
- Kien Quoc Do is responsible for study design, data collection, analysis, manuscript development, and dissemination of study findings.
- Kien Quoc Do is responsible for contacting researchers from Griffith University, University of Queensland, and Pasteur Institute in Ho Chi Minh City to set up an interviewing team. Kien Quoc Do also provides training to inform the activities of routine dengue surveillance and responses, and to conduct the in-depth interviews.
- Kien Quoc Do invite independent expert in qualitative study and dengue prevention and response for the coding process.
- Ha Thu Nguyen is responsible for data collection, the verbatim transcript, member checking, manuscript review, and editing.
- Thi Thanh Thao Nguyen is responsible for the verbatim transcript, member checking, manuscript review, and editing.
- National Institute of Hygiene and Epidemiology, led by Thinh Nguyen, is responsible for coordinating activities, investigating, and collecting data.
- CDCs of An Giang and Long An are operating the breeding source reduction and scheduling vector investigation and data collection.
- The UQ team is responsible for posting and communicating study findings.

*Expected deliverables*

- Evidence depicting the barriers to the proactive source reduction informed by EWS.
- Manuscript disseminating study findings.
