# Supplementary material for: Barriers in operating proactive source reduction informed by an early warning system for dengue prevention: A qualitative study from end-user perspectives in Vietnam
Source: PLoS Negl Trop Dis. 2026 Sep 25;20(9):e0014754. doi: 10.1371/journal.pntd.0014754 (PMC13649133; doi:10.1371/journal.pntd.0014754)
Supplement: Supplementary file 8 [file pntd.0014754.s008.docx]

**Reviewer Comments**

**Recommendation: Major Revision**

**Major Comments**

1. **Novelty unclear**
   The manuscript does not clearly distinguish barriers specific to *EWS-informed interventions* from general dengue control barriers. Please explicitly state the unique contribution, particularly regarding challenges of using probabilistic EWS outputs in decision-making.
2. **Analysis too descriptive**
   Findings are mainly descriptive. Add interpretive synthesis showing how barriers interact (e.g., policy → authority → implementation → community).
3. **EWS description insufficient**
   Provide more detail on E-Dengue:
   - What outputs/alerts look like
   - How they are communicated
   - How they trigger action
4. **Methods need clarification**
   - Clarify deductive vs inductive coding
   - Describe translation (Vietnamese → English)
   - Better justify small sample (n=15) and saturation claim
5. **Data availability not compliant**
   “Available upon request” is insufficient. Clarify what data can be shared or strengthen justification for restriction.
6. **Overgeneralization**
   Tone down claims such as “transferable lessons” given limited, pilot-based data.

**Minor Comments**

- Revise title: “Barriers to implementing…”
- Correct typo: “lavivorous” → “larvivorous”
- Reduce repetition of “informed by EWS”
