# Supplementary material for: Barriers in operating proactive source reduction informed by an early warning system for dengue prevention: A qualitative study from end-user perspectives in Vietnam
Source: PLoS Negl Trop Dis. 2026 Sep 25;20(9):e0014754. doi: 10.1371/journal.pntd.0014754 (PMC13649133; doi:10.1371/journal.pntd.0014754)
Supplement: Supplementary file 9 [file pntd.0014754.s010.docx]

Minor concern/revision

- As article talk about barriers from end-user perspectives including community but there is perspective via proxy through other representatives like health volunteers or community health station member. However, there is not direct representatives from household members to know end-user barriers in implementation. So, explain the limitation of respondent in discussion section.
- In line 308 and 342, the number of quotations under the theme “Operating source reduction at the community level” and “community perception” is more in number. Just keep the quotation as much as limited which are more context to the theme.
