# Supplementary material for: Barriers in operating proactive source reduction informed by an early warning system for dengue prevention: A qualitative study from end-user perspectives in Vietnam
Source: PLoS Negl Trop Dis. 2026 Sep 25;20(9):e0014754. doi: 10.1371/journal.pntd.0014754 (PMC13649133; doi:10.1371/journal.pntd.0014754)
Supplement: Supplementary file 10 [file pntd.0014754.s011.docx]

**Response to Editor and Reviewers**

**Manuscript title:** Barriers in operating proactive source reduction informed by an early warning system for dengue prevention: A qualitative study from end-user perspectives in Vietnam.

**PNTD-D-26-00374**

We thank the editor and reviewers for your thorough review and provide further recommendations and comments.
This letter provides a point-by-point response to each reviewer's questions and comments, indicating how they have been addressed.

**Editor comments**

| **Comments** | **Responses** | **Action taken** |
| --- | --- | --- |
| The study remains largely descriptive and does not sufficiently elevate its findings to a conceptual or theoretical level. | We acknowledge that, as presented in the original manuscript, the findings may have appeared largely descriptive. Rather than generating a new theory, we intended to refine and extend the existing conceptual framework by examining how multilevel barriers interact within a real-world implementation context, thereby providing transferable insights for other dengue-endemic settings. This study was designed to identify barriers to implementing EWS-informed proactive source reduction, a novel approach to dengue prevention and control that has not been routinely integrated into dengue programs in Vietnam and other countries. The study was informed by and sought to extend existing conceptual frameworks developed from previous research in Vietnam and Malaysia regarding the implementation of dengue early warning systems. | We have strengthened the manuscript by providing a clearer description of the methodological approach and by expanding the analytical interpretation of the findings beyond a descriptive thematic presentation. Specifically, we further explored the interrelationships among barriers at the policy, operational, and community levels and discussed how these barriers collectively influence the translation of EWS alerts into proactive vector control actions. These revisions aim to elevate the findings from a descriptive account towards a more conceptual understanding of the factors shaping EWS implementation in dengue-endemic settings, similar to Vietnam context. |
| The contribution is currently more implicit than clearly positioned within the broader implementation of science or health systems literature. | We agree that the contribution of the study was not sufficiently articulated in the original manuscript and may therefore have appeared implicit rather than clearly positioned within the broader implementation of science and health systems literature. | In the revised version, we have expanded the discussion of the mechanism leading to each barrier, as recommended by reviewers, to better contextualize our findings within existing implementation science frameworks and to emphasize how multilevel barriers influence the integration of EWS-informed interventions into routine dengue control programs. These revisions clarify the broader relevance of our findings for implementing data-driven public health interventions in dengue-endemic and resource-constrained settings. |

**Reviewer #1:**

| **Comments** | **Responses** | **Action taken** |
| --- | --- | --- |
| **Methods** | | |
| The study objectives are not explicitly framed within a well-defined conceptual or theoretical framework. The manuscript would benefit from a clearer articulation of its analytical aims (e.g., whether it seeks to generate, extend, or test an implementation framework). Strengthening this aspect would improve coherence between objectives, methods, and interpretation. | We agree that clearer articulation of conceptual framing and analytical aims would strengthen the coherence between the study objectives, methods, and interpretation. This study was conducted and further expanded based on a predefined conceptual framework developed from previous studies in Vietnam and Malaysia that examined barriers, operational challenges, and community needs related to the implementation of dengue source reduction activities. Building on this framework with further expansion during the analytic process, our study aimed to explore how these factors influence the integration and operationalization of EWS-informed proactive interventions. | We have revised the manuscript to further describe the use and expansion of a predefined conceptual framework across the Introduction (Page 3, lines 105 and 106), study design (Page 5, lines 156 and 165), coding and analysis (Page 8, lines 251 to 272), and results (Page 9, lines 293 to 298) sections. The revised text explicitly states that the study was guided by a predefined conceptual framework, which was subsequently refined and expanded based on empirical findings from the interviews. |
| The population is limited to formal system actors, with insufficient representation of community-level participants, particularly individuals from higher- and lower-risk populations. This restricts the ability to capture lived experiences and limits triangulation across perspectives. | We acknowledge that the limited inclusion of participants directly from community members, particularly individuals from higher- and lower-risk populations, may restrict the ability to fully capture lived experiences and limit triangulation across perspectives. Although our study included interviews with health volunteers (HVs), who commonly serve as representatives and close liaisons with the communities they support, we recognize that their perspectives may not fully reflect the diversity of community experiences and perceptions. | We added further information describing the role of HVs in Vietnam, who commonly serve as representatives and close liaisons for the communities they support in the study design section (Page 6, lines 186-187 and 201-205). We also acknowledged the limitation of lacking community members in the revised manuscript, expanded the discussion on the limitations related to community representation, and highlighted the need for future studies to include broader community participation to strengthen contextual understanding and triangulation across perspectives (Page 19, lines 605 to 615). |
| The manuscript does not clearly describe how sampling was conducted, the size of the eligible population, or how data saturation was assessed. | All health staff and Commune People’s Committee leaders who were involved in the pilot implementation of the E-Dengue project were invited and recruited. In addition, Commune Health Station (CHS) staff and HVs were recruited using a purposive sampling approach guided by the principle of data saturation. The assessment of data saturation and the emergence of new information among CHS staff and HVs were conducted iteratively throughout data collection. After each interview, the research team completed a checklist for interview summary to document emerging themes, recurring issues, and the extent of new information generated. Recruitment of CHS staff and HVs continued until no substantial new themes or insights emerged from subsequent interviews. These details have now been added to the methods section to improve transparency regarding participant recruitment and sampling adequacy. | We have revised the study design section (Pages 5 and 6, lines 167 to 209) to provide additional details clarifying that all health staff across all levels, as well as Commune People’s Committee leaders directly involved in the pilot implementation of the E-Dengue project, were invited and recruited for participation. We also added a more detailed description of the procedures used to monitor data saturation among CHS staff and HVs, including the iterative assessment of emerging themes and new information during data collection. In addition, we have provided a sample checklist used by the research team to monitor data saturation and interview completeness (Supplement 2). |
| The manuscript does not sufficiently explain coding procedures, inter-researcher consistency, or the balance between inductive and deductive approaches. Additional clarification is needed to demonstrate rigor and reproducibility. No new statistical analyses are required, but a more explicit and transparent analytical process is necessary. | Data was analysed using thematic analysis, following the Braun and Clarke framework, which employed a hybrid approach to coding. We initially adopted a deductive approach to develop a foundational codebook informed by the predefined conceptual framework from studies in Vietnam and Malaysia. To maintain analytical openness, inductive coding was subsequently incorporated to capture emerging concepts that were not represented in the initial framework. The initial codes were predefined by the conceptual framework and refined during familiarisation with the transcripts. The analyst independently coded all transcripts line-by-line using ATLAS.ti software (version 25.0). Code and theme structures were discussed with the research team to ensure consistency in code interpretation and application across transcripts. Coding discrepancies and ambiguous interpretations were reviewed and resolved, with the codebook updated accordingly throughout the analytical process. Themes were iteratively generated by grouping conceptually related codes and were continuously refined through iterative comparison across participant groups and interview transcripts. New themes emerging inductively from the data were incorporated when concepts were not adequately represented within the initial coding framework. Theme definitions were subsequently revised, consolidated, and conceptually distinct themes were separated to ensure that the final themes accurately reflected the underlying data and maintained analytical consistency. | We have revised the “Coding and Analysis” section (Page 8, lines 251 to 278) to provide a more detailed description of the coding procedures, including how codes were developed and refined, the balance between deductive coding based on the predefined conceptual framework and inductive coding from emerging interview data, as well as the processes used to ensure consistency among researchers during coding and interpretation. |
| The manuscript should specify whether informed consent was written or oral and how it was documented and approved. The data availability statement may not fully comply with PLOS policies and should be revised to ensure transparency and accessibility or to clearly justify restrictions. | Prior to each interview, participants were informed about the study objectives, a summary of the study, interview procedures, and their rights to refuse participation or withdraw from the study at any time without consequences. Opportunities were provided for participants to discuss the study and ask questions with the interviewers before participation. Interviews were conducted only after written informed consent had been obtained. With participants’ consent, interviews were audio-recorded with concurrent note-taking. | We have revised the manuscript to provide additional details regarding the informed consent process in the data collection section (Page 7, lines 231 to 238) for further and clearer clarification, as described in our response. We also provided the supplement of our information and consent form to support our description (Supplement 4). In addition, we reviewed the PLOS data sharing requirements and revised the data availability statement accordingly to ensure better compliance with the journal’s policies (Page 20, lines 658 to 663). |
| **Results** | | |
| The linkage between the analytical framework and the actual coding and theme development process is not fully transparent. In particular, it remains unclear how themes were derived, whether they emerged inductively from the data or were primarily guided by a pre-existing framework, and how consistency in coding was ensured across researchers. Clarifying this alignment would strengthen confidence in the analytical rigor. | We initially adopted a deductive approach to develop a foundational codebook informed by the predefined conceptual framework. To maintain analytical openness, inductive coding was subsequently incorporated to capture emerging concepts that were not represented in the initial framework, in which the initial codes were predefined by the conceptual framework and refined during familiarisation with the transcripts, while additional codes and themes were allowed to emerge from the data. Inductive themes emerged during analysis, particularly relating to the lack of supportive policies, inadequate local authority support, operational challenges in implementing source reduction at the community level, community perceptions, and poorly adaptable intervention measures. | We have revised the Methods section to provide a more transparent description of the coding and analyses (Page 8, lines 251 to 276). Specifically, we now describe the use of a hybrid deductive–inductive thematic analysis approach, whereby an initial codebook was developed from the predefined conceptual framework and subsequently refined through inductive coding to capture concepts emerging from the data. We also provide additional details on how codes were generated, how themes and subthemes were derived through iterative comparison and refinement, and how coding consistency was ensured through regular review and discussion of coding decisions and theme development within the research team.  In addition, to improve transparency in the presentation of findings, we replaced the Sankey diagram with a hierarchical table that more clearly illustrates the relationships between themes, subthemes, and representative codes identified through the thematic analysis (Page 10, lines 294 to 315). |
| The analysis remains largely descriptive and would benefit from deeper analytical interpretation. Specifically, the manuscript could more explicitly examine underlying mechanisms (i.e., why barriers occur), interactions across system levels, and potential divergences in perspectives among stakeholder groups. In addition, the absence of community-level participants limits the completeness of the findings and reduces opportunities for triangulation with lived experiences. | We agree that the results section was primarily focused on describing the identified themes and barriers, while more detailed interpretation of the underlying mechanisms and interactions across system levels was mainly elaborated in the discussion section. We acknowledge that providing additional explanation of how and why these barriers emerged within the results section would help readers better understand the relationships between themes and improve the linkage between the results and discussion sections, as suggested by the reviewer. | We revised the results section to include a more in-depth description of the mechanisms underlying the identified barriers, interactions across different levels of the health system, and areas of convergence or divergence among stakeholder perspectives where relevant (Pages 11 and 12; lines 317 to 333, page 12, lines 350 to 359; page 13, lines 379 to 386; page 14, lines 415 to 419; page 15, lines 464 to 471). We also acknowledged that the absence of broader community-level participants may limit the completeness of triangulation with lived experiences, and this limitation has been further discussed in the discussion section (Pages 17 and 18, lines 561 to 577) of the revised manuscript. |
| Tables summarizing themes and subthemes would benefit from clearer linkage to the analytical framework and, where possible, more explicit illustration of relationships between categories (e.g., hierarchical or relational structures). | We agree that the previously provided figure did not sufficiently illustrate the linkage to the analytical framework or the relationships between categories identified in the analysis. | We revised the presentation of the findings by replacing the Figure 2 with a table that more clearly describes the hierarchical structure of themes and subthemes identified through thematic analysis (Pages 10 and 11, lines 304 to 309). This revision was intended to improve the transparency and coherence of the analytical framework and the relationships between categories. |
| **Conclusions** | | |
| Greater caution is warranted to ensure that conclusions are clearly framed as context-specific, evidence-informed interpretations derived from qualitative insights rather than broadly generalizable claims. | We agree that greater caution is needed to ensure that the conclusions are appropriately framed as context-specific and evidence-informed interpretations derived from qualitative findings, rather than broadly generalizable claims. As this qualitative study was conducted within the specific health system and sociocultural context of Vietnam, the findings should be interpreted with consideration of local organisational structures, community practices, and dengue prevention strategies. Therefore, the applicability of these findings may be more relevant to settings with social, operational, and public health contexts similar to those in Vietnam, rather than being generalized broadly across all dengue-endemic settings. | We have revised the abstract conclusions (Page 2, lines 39 to 49), author summary (Page 2, line 69), and the conclusions (Pages 19 and 20, lines 638 to 658) accordingly to better reflect this contextual interpretation. |
| The manuscript would benefit from a more explicit and comprehensive discussion of its limitations. While some constraints are implicit, key issues are not sufficiently addressed, including: (i) the absence of community-level participants, which limits triangulation and the representation of lived experiences; (ii) potential sampling constraints and transferability; and (iii) the influence of contextual and cultural factors (e.g., Vietnamese sociocultural dynamics) that may shape both implementation barriers and interpretation of findings. A clearer articulation of these limitations would strengthen the transparency and credibility of the study. | We agree that the limitations highlighted by the reviewer may have influenced the study findings and warrant more explicit discussion to strengthen the transparency and credibility of the manuscript. These issues need further discussion regarding their potential impact on the findings, as well as the abilities of approaches used to minimise their influence on data collection, analysis, and interpretation. | We have revised the discussion and added further clarification regarding the potential impacts and abilities of approaches used to mitigate the potential impacts of these factors, as highlighted by the reviewer, in the discussion section (Pages 18 and 19, lines 599 to 636). |
| The discussion could more strongly articulate how these findings advance conceptual understanding. In its current form, the manuscript tends to confirm existing knowledge rather than clearly extending it. Greater engagement with implementation science or health systems frameworks, and the articulation of a more explicit conceptual contribution (e.g., a model of multi-level implementation barriers), would enhance its scientific value. | We agree that the original discussion section placed greater emphasis on describing and confirming previously reported barriers, while the conceptual contribution of the study was not sufficiently articulated. | We revised the discussion to more explicitly describe how the findings extend existing understanding of EWS-informed dengue prevention from an implementation and health systems perspective. Particularly, we strengthened the discussion on how barriers interact across multiple levels, including governance and policy, organisational and operational systems, risk communication, and community perceptions and practices (Pages 17 and 18, lines 561 to 577). We also clarified the study’s conceptual contribution by describing how the findings support and further refine a multi-level implementation-oriented framework for operationalising EWS-informed proactive dengue prevention within routine health system practice (Page 18, lines 582 to 598). |
| Strengthening the linkage between the results and broader, multi-component dengue prevention strategies (including individual-level protective behaviors) would further enhance its relevance and alignment with global public health frameworks. | We agree that additional information strengthening the linkage between the results and broader, multi-component dengue prevention strategies would further enhance the relevance of the manuscript and its alignment with global public health frameworks. Our findings highlight that EWS-informed proactive source reduction represents a novel dengue prevention approach that differs from conventional strategies primarily triggered by index cases and outbreak responses. The implementation of this approach was shaped by interactions across governance, operational, and community levels, including stakeholder coordination, the translation of early warning information into actionable interventions, and community perceptions regarding dengue risk and preventive behaviours. These findings further emphasize that successful operationalisation of EWS-informed proactive dengue prevention requires integration within broader health system and community-based prevention strategies rather than relying solely on predictive accuracy or vector control activities alone. | We have added an additional paragraph on pages 17 and 18, lines 561 to 577 to further elaborate on these issues, as suggested by the reviewer. |
| **Editorial and Data Presentation Modification** | | |
| A careful, line-by-line language revision by a proficient English speaker or professional editing service is strongly recommended to ensure accuracy and coherence throughout the manuscript. | We acknowledge this oversight during the writing process. | The manuscript has been reviewed and revised to ensure consistent and appropriate tense usage throughout the text. |
| Some sentences require minor restructuring to improve clarity and precision (e.g., incomplete clauses, inconsistent use of articles, or ambiguous phrasing). | The sentences identified by the reviewer have been carefully checked and revised to improve clarity and precision. In addition, we conducted a thorough review of the entire manuscript to identify and correct any similar issues. | The manuscript has been reviewed and revised to ensure consistent and appropriate tense usage throughout the text. |
| The current data availability statement is inconsistently phrased across sections and may not fully align with PLOS requirements. The statement should be standardized and revised to clearly specify whether data are publicly available, conditionally accessible, or restricted, including a transparent process for access where applicable. | We acknowledge that the Data Availability Statement was not presented consistently across the manuscript and may not have fully aligned with PLOS requirements. | We have revised and standardized the statement throughout the manuscript to clearly specify the availability status of the study data (Page 20, lines 660 to 665). |
| The reference list requires a comprehensive editorial revision to ensure full compliance with Vancouver style. | The reference list was generated using Zotero; however, some formatting inconsistencies may have originated from the source data. | We have reviewed the references and manually corrected entries that did not conform to Vancouver style to ensure consistency and compliance with the journal requirements. |
| Tables and figures are generally adequate but could be improved to enhance interpretability. In particular, tables summarizing themes and subthemes would benefit from clearer labeling and stronger alignment with the analytical framework. Where possible, figures or tables could more explicitly reflect relationships between themes rather than presenting purely descriptive categorizations. | We agree that the original Sankey diagram primarily presented descriptive categorizations and did not sufficiently illustrate the relationships between codes, subthemes, and overarching themes within the analytical framework. | The Figure 2 was replaced by a hierarchical table to more clearly represent conceptual linkages, thereby improving the interpretation of how individual codes contributed to subthemes and themes (Pages 10 and 11, lines 304 to 309). |

**Reviewer #2:**

| **Comments** | **Responses** | **Action taken** |
| --- | --- | --- |
| **Methods** | | |
| Supplement the start and end months of the rainy season in the pilot provinces, local water supply systems, and primary behaviors of residents in responding to water scarcity to align with the content in the Results and Discussion sections. | We agree that providing additional contextual information regarding seasonal patterns, water supply systems, and household water storage practices would improve the interpretation and coherence of the results and discussion sections. Dengue transmission in the study provinces is characterized by seasonal transmission patterns commonly observed in tropical countries, with increasing transmission during the rainy season from June to November. In addition, household water storage was commonly practiced in the study areas due to concerns regarding limited water supply, as well as preferences for rainwater storage based on perceptions of lower cost, better water quality, and greater supply security for daily use. | We have supplemented the manuscript with additional descriptions in the study settings section (page 4) indicating that dengue transmission in these provinces is characterized by seasonal transmission patterns, with increasing transmission from June to November (Page 4, line 129). We also added information describing household water storage practices in the study provinces (Page 4, lines 130 to 133). Additional relevant references were provided to further support these contextual descriptions and justifications. |
| Please provide additional details regarding the primary organizational implementation of the pilot project. | The pilot implementation was administratively managed by the Departments of Health (DHs), with technical guidance and monitoring provided by the Centres for Disease Control (CDCs). Based on EWS alerts, the CDCs coordinated with Regional Health Centres (RHCs) to develop implementation plans and organize source reduction activities. The RHCs subsequently collaborated with Commune People’s Committees (CPCs) and Commune Health Stations (CHSs) to mobilize health volunteers (HVs) and engage other relevant stakeholders. Source reduction activities were then delivered at the household level through HV household visits under the coordination of CHSs and the supervision of RHCs and CDCs.  In this study, we applied the national source reduction implementation procedure in which households were visited by health volunteers to provide dengue health education and encourage larval source reduction practices for dengue prevention. This routine source reduction process was informed and triggered by the Early Warning System (EWS). | We have added further details describing the implementation procedures and organisational implementation of the project as suggested in the study settings section (Page 4, lines 138 to 148). |
| In the Study design section, please elaborate on how the samples from the two pilot provinces were allocated. What are the differences in limitations regarding purposive sampling methods applied to interviewees across six levels? | In our study, all health staff and Commune People’s Committee leaders who were involved in the pilot implementation of the E-Dengue project were invited and recruited. In addition, HVs were recruited using a purposive sampling approach guided by the principle of data saturation. The assessment of data saturation and the emergence of new information among HVs was conducted iteratively throughout data collection. After each interview, the research team completed a checklist for interview summary to document emerging themes, recurring issues, and the extent of new information generated. Recruitment of HVs continued until no substantial new themes or insights emerged from subsequent interviews. | We have revised the Study Design section (Pages 5 and 6, lines 172 to 188) to provide additional details clarifying that all health staff across all levels, as well as Commune People’s Committee leaders directly involved in the pilot implementation of the E-Dengue project, were invited and recruited for participation. We also added a more detailed description of the procedures used to monitor data saturation among CHS staff and HVs, including the iterative assessment of emerging themes and new information during data collection (Page 6, lines 189 to 209).  We also revised the discussion section to further acknowledge and elaborate on the limitations associated with this sampling approach (Pages 18 and 19, lines 599 to 623). |
| **Conclusions** | | |
| The conclusions are supported by the results, and the limitations should be modified by adding some discussion about the study sampling in the two pilot sites. | We agree that the relatively small sample size, the inclusion of participants from only two pilot sites, and the absence of community-level participants may have influenced the study findings and therefore warrant further discussion in the manuscript. | We expanded the discussion section (Pages 18 and 19, lines 599 to 623) to further acknowledge these sampling-related limitations, including their potential impact on the breadth and transferability of perspectives captured in the study. We also discussed the contextual nature of the findings and the need for future studies involving broader community representation and diverse implementation settings (Page 19, lines 624 to 636). |

**Reviewer #3:**

| **Comments** | **Responses** | **Action taken** |
| --- | --- | --- |
| **Methods** | | |
| Clarify how deductive coding was combined with thematic analysis—specifically, how initial codes were defined and how new themes were generated and refined. | We initially adopted a deductive approach to develop a foundational codebook informed by the predefined conceptual framework from studies in Vietnam and Malaysia. To maintain analytical openness, inductive coding was subsequently incorporated to capture emerging concepts that were not represented in the initial framework. The initial codes were predefined by the conceptual framework and refined during familiarisation with the transcripts. The analyst independently coded all transcripts line-by-line using ATLAS.ti software (version 25.0). Code and theme structures were discussed with the research team to ensure consistency in code interpretation and application across transcripts. Coding discrepancies and ambiguous interpretations were reviewed and resolved, with the codebook updated accordingly throughout the analytical process. Themes were iteratively generated by grouping conceptually related codes and were continuously refined through iterative comparison across participant groups and interview transcripts. New themes emerging inductively from the data were incorporated when concepts were not adequately represented within the initial coding framework. Theme definitions were subsequently revised, consolidated, and conceptually distinct themes were separated to ensure that the final themes accurately reflected the underlying data and maintained analytical consistency. | We have revised the “Coding and Analysis” section (Page8, lines 251 to 276) to provide a more detailed description of the coding procedures, including how codes were developed and refined, the balance between deductive coding based on the predefined conceptual framework and inductive coding from emerging interview data, as well as the processes used to ensure consistency among researchers during coding and interpretation. |
| **Conclusions** | | |
| The conclusion overgeneralizes findings; revise to reflect the limited, context-specific nature of the study and avoid broad claims of transferability. | We agree that the original conclusion overgeneralized the findings beyond the analytical evidence generated from this context-specific qualitative study. | We revised the abstract conclusions (Page 2, lines 39 to 49), author summary (Page 2, line 69), and conclusions (Pages 19 and 20, lines 638 to 656) to more clearly frame the findings as context-specific and evidence-informed interpretations derived from the implementation experiences within the Vietnamese health system. We also clarified that the applicability of the findings may be more relevant to settings with similar organisational, operational, and sociocultural contexts. |

**Reviewer #4:**

| **Comments** | **Responses** | **Action taken** |
| --- | --- | --- |
| **Methods** | | |
| "Data saturation" is a key concept in the interview analysis. The authors should elaborate on the formal saturation analysis performed (i.e., through the inclusion of assessment criteria). | The assessment of data saturation and the emergence of new information among HVs was conducted iteratively throughout data collection. After each interview, the research team completed a checklist for interview summary to document emerging themes, recurring issues, and the extent of new information generated. Recruitment of CHS staff and HVs continued until no substantial new themes or insights emerged from subsequent interviews. | We added a more detailed description of the procedures used to monitor and obtain data saturation among HVs, including the iterative assessment of emerging themes and new information during data collection (Page 6, lines 189 to 209). In addition, we have provided a sample checklist used by the research team to monitor data saturation and interview completeness (Supplement 2). |
| The sample size of 15 across six different stakeholder levels is acceptable, yet relatively thin. The authors should explicitly discuss how the sample size might impact the study results. | We acknowledge that the relatively small sample size may have limited the breadth of perspectives captured and potentially influenced the findings, and this issue warrants further discussion in the manuscript. In this study, we included all health staff and local authority representatives who were directly involved in the E-Dengue pilot implementation across the two study provinces. In addition, most health professionals had substantial experience in dengue prevention and control, ranging from 2 to 23 years (Table 1), which likely strengthened their ability to identify and describe barriers encountered during routine dengue prevention practices in their local settings. | We have further elaborated on these considerations in the revised discussion section as a study limitation (Pages 18 and 19, lines 599 to 623). |
| **Results** | | |
| Ensure that Figure 2 (Thematic Framework) is provided in high resolution, as the conceptual flow is vital for the reader's understanding. Currently, the words in Fig 2 were relatively hard to recognize, unless the reader zoom in. | We acknowledge that the text and conceptual flow presented in Figure 2 were difficult to recognize and interpret without zooming in. We also agreed with another reviewer’s comment that the figure did not adequately illustrate the relationships between categories identified in the analysis. Therefore, we replaced Figure 2 with a table that more clearly describes the hierarchical structure of themes and subthemes identified through thematic analysis, thereby improving readability and transparency of the analytical framework. | We have replaced Figure 2 with a table describing the hierarchical structure of themes and subthemes identified through thematic analysis (Pages 10 and 11, lines 304 to 315). |
| **Conclusions** | | |
| The conclusion section clearly states the novelty and limitations of the research. However, the conclusion will be considered stronger if a more rigorous data analysis were performed. | We agree that, in the original version, the analytical procedures were not described in sufficient detail to allow readers to fully understand the rigor of the data analysis process. Rigorous qualitative analysis was ensured through the use of thematic analysis following the Braun and Clarke framework, employing a hybrid deductive–inductive coding approach. We initially adopted a deductive approach to develop a foundational codebook informed by the predefined conceptual framework derived from previous studies conducted in Vietnam and Malaysia. To maintain analytical openness, inductive coding was subsequently incorporated to capture emerging concepts not represented within the initial framework. The initial codes were refined during transcript familiarisation, and all transcripts were independently coded line-by-line. Code definitions, coding interpretations, and theme structures were regularly discussed among the research team to ensure consistency and analytical coherence across transcripts. Coding discrepancies and ambiguous interpretations were reviewed and resolved iteratively, with the codebook updated throughout the analysis process. Themes were generated through iterative grouping and comparison of conceptually related codes across participant groups and interview transcripts. Emerging inductive themes were incorporated where necessary, and theme definitions were continuously refined and consolidated to ensure conceptual distinction and accurate representation of the underlying data. | We have revised the “Coding and Analysis” section (Page 8, lines 251 to 276) to provide a more detailed description of the coding procedures, including how codes were developed and refined, the balance between deductive coding based on the predefined conceptual framework and inductive coding from emerging interview data, as well as the processes used to ensure consistency among researchers during coding and interpretation. |
| **Editorial and Data Presentation Modification** | | |
| There is a constant typo of "Barries" in the uploaded file name. | Thank you for noting this error. "Barries" was a typographical mistake in the file name. This has been corrected in the revised version for resubmission. | We have reviewed and renamed the uploaded files to correct the typographical mistake. |
| The study mentions interviews in "September to October 2025". Given the current date is March 2026, the authors should ensure all tense usage (past vs. present) is consistent. | We acknowledge this oversight during the writing process. | The manuscript has been reviewed and revised to ensure consistent and appropriate tense usage throughout the text. |
