# Supplementary material for: Barriers in operating proactive source reduction informed by an early warning system for dengue prevention: A qualitative study from end-user perspectives in Vietnam
Source: PLoS Negl Trop Dis. 2026 Sep 25;20(9):e0014754. doi: 10.1371/journal.pntd.0014754 (PMC13649133; doi:10.1371/journal.pntd.0014754)
Supplement: Peer review history

# Peer review: Decision Letter 0

**DOI:** 10.1371/journal.pntd.0014754.r001

**Authors:** Luis Diaz, Victoria Brookes

15 Apr 2026

Barriers in operating proactive source reduction informed by an early warning system for dengue prevention: A qualitative study from end-user perspectives in Vietnam

PLOS Neglected Tropical Diseases

Dear Dr. Do,

Thank you for submitting your manuscript to PLOS Neglected Tropical Diseases. After careful consideration, we feel that it has merit but does not fully meet PLOS Neglected Tropical Diseases's publication criteria as it currently stands. Therefore, we invite you to submit a revised version of the manuscript that addresses the points raised during the review process.

Please submit your revised manuscript within by Jun 14 2026 11:59PM. If you will need more time than this to complete your revisions, please reply to this message or contact the journal office at plosntds@plos.org. When you're ready to submit your revision, log on to https://www.editorialmanager.com/pntd/ and select the 'Submissions Needing Revision' folder to locate your manuscript file.

Please include the following items when submitting your revised manuscript:

* A letter that responds to each point raised by the editor and reviewer(s). You should upload this letter as a separate file labeled 'Response to Reviewers'. This file does not need to include responses to any formatting updates and technical items listed in the 'Journal Requirements' section below.

* A marked-up copy of your manuscript that highlights changes made to the original version. You should upload this as a separate file labeled 'Revised Manuscript with Track Changes'.

* An unmarked version of your revised paper without tracked changes. You should upload this as a separate file labeled 'Manuscript'.

If you would like to make changes to your financial disclosure, competing interests statement, or data availability statement, please make these updates within the submission form at the time of resubmission. Guidelines for resubmitting your figure files are available below the reviewer comments at the end of this letter.

We look forward to receiving your revised manuscript.

Kind regards,

Luis Adrián Diaz, Ph.D.

Academic Editor

PLOS Neglected Tropical Diseases

Victoria Brookes

Section Editor

PLOS Neglected Tropical Diseases

Shaden Kamhawi

co-Editor-in-Chief

PLOS Neglected Tropical Diseases

orcid.org/0000-0003-4304-636XX

Paul Brindley

co-Editor-in-Chief

PLOS Neglected Tropical Diseases

orcid.org/0000-0003-1765-0002

**Additional Editor Comments:**

Dear Authors,

Thank you very much for considering PLOS Neglected Tropical Diseases as a venue to disseminate your work. Your manuscript has been evaluated by four specialists in the field, all of whom recognized the quality and importance of your research.

The manuscript addresses a relevant and operationally important topic within our scope, and the qualitative design is appropriate. The study provides useful multi-level insights from stakeholders involved in dengue prevention in Vietnam, and the manuscript is generally well-structured and clearly written.

However, in its current form, the manuscript does not yet reach the level of conceptual and analytical depth expected for a Q1 journal. The main limitation is that the study remains largely descriptive and does not sufficiently elevate its findings to a conceptual or theoretical level. The contribution is currently more implicit than clearly positioned within the broader implementation science or health systems literature.

Importantly, the issues identified are not related to fundamental flaws in study design or data integrity, but rather to analytical depth, conceptual framing, and reporting transparency. These are substantial but addressable through revision. Therefore, I am recommending a Major Revision, as the manuscript would benefit from:

Clearer articulation of its conceptual contribution and positioning within the literature.

Greater analytical depth, moving beyond descriptive thematic reporting.

Improved methodological transparency (sampling, saturation, and coding procedures).

Clarification of ethical reporting (informed consent and data availability compliance).

I do not see grounds for rejection at this stage, as the study has clear practical relevance and a solid qualitative foundation. However, acceptance will be contingent on a meaningful revision that strengthens the manuscript’s theoretical contribution and analytical rigor. Please also ensure that minor issues related to reporting transparency, such as data availability statement consistency, are addressed.

Please provide a point-by-point response to both the editorial comments above and the specific reviewers' remarks attached below.

Sincerely,

**Journal Requirements:**

1) Please upload all main figures as separate Figure files in .tif or .eps format. For more information about how to convert and format your figure files please see our guidelines:

https://journals.plos.org/plosntds/s/figures

2) We have noticed that you have uploaded Supporting Information files, but you have not included a list of legends. Please add a full list of legends for your Supporting Information files after the references list.

3) In the online submission form, you indicated that "Data may be made available upon reasonable request and with approval from the Ethics Committee of the National Institute of Hygiene and Epidemiology in Vietnam, in accordance with institutional regulations.". All PLOS journals now require all data underlying the findings described in their manuscript to be freely available to other researchers, either

1. In a public repository

2. Within the manuscript itself

3. Uploaded as supplementary information.

This policy applies to all data except where public deposition would breach compliance with the protocol approved by your research ethics board. If your data cannot be made publicly available for ethical or legal reasons (e.g., public availability would compromise patient privacy), please explain your reasons by return email and your exemption request will be escalated to the editor for approval. Your exemption request will be handled independently and will not hold up the peer review process, but will need to be resolved should your manuscript be accepted for publication. One of the Editorial team will then be in touch if there are any issues.

**Reviewers' Comments:**

Reviewer's Responses to Questions

**Key Review Criteria Required for Acceptance?**

As you describe the new analyses required for acceptance, please consider the following:

**Methods**

-Are the objectives of the study clearly articulated with a clear testable hypothesis stated?

-Is the study design appropriate to address the stated objectives?

-Is the population clearly described and appropriate for the hypothesis being tested?

-Is the sample size sufficient to ensure adequate power to address the hypothesis being tested?

-Were correct statistical analysis used to support conclusions?

-Are there concerns about ethical or regulatory requirements being met?

Reviewer #1: 1. Key Review Criteria Required for Acceptance

The manuscript addresses a relevant and timely public health problem, and the overall qualitative design is appropriate. However, several issues require attention before the study can be considered for publication.

Methods and Objectives

The study objectives are generally clear; however, they are not explicitly framed within a well-defined conceptual or theoretical framework. While qualitative studies do not require “testable hypotheses” in the traditional sense, the manuscript would benefit from a clearer articulation of its analytical aims (e.g., whether it seeks to generate, extend, or test an implementation framework). Strengthening this aspect would improve coherence between objectives, methods, and interpretation.

Study Design and Population

The qualitative design is appropriate for exploring implementation barriers. The inclusion of multi-level institutional stakeholders is a strength. However, the population is limited to formal system actors, with insufficient representation of community-level participants, particularly individuals from higher- and lower-risk populations. This restricts the ability to capture lived experiences and limits triangulation across perspectives.

Sample Size and Saturation

Although qualitative adequacy is implied, the manuscript does not clearly describe how sampling was conducted, the size of the eligible population, or how data saturation was assessed. Greater transparency is required to evaluate the robustness and coverage of the sample.

Analytical Approach

The thematic analysis is appropriate, but the analytical depth remains largely descriptive. The manuscript does not sufficiently explain coding procedures, inter-researcher consistency, or the balance between inductive and deductive approaches. Additional clarification is needed to demonstrate rigor and reproducibility. No new statistical analyses are required, but a more explicit and transparent analytical process is necessary.

Ethical and Regulatory Considerations

Ethical aspects are addressed but require clarification. In particular, the manuscript should specify whether informed consent was written or oral and how it was documented and approved. The data availability statement may not fully comply with PLOS policies and should be revised to ensure transparency and accessibility or to clearly justify restrictions.

Summary of Required Revisions

The manuscript does not require new experiments or quantitative analyses, but it does require several substantial methodological and analytical clarifications and expansions, including:

1. Clearer conceptual framing and articulation of analytical objectives.

2. Expanded description and justification of sampling procedures and data saturation.

3. Greater transparency and rigor in the analytical (coding) process.

4. Improved representation or discussion of community-level perspectives and triangulation limitations.

5. Clarification of ethical procedures and data availability compliance.

Recommendation

Given that the required revisions are substantial, I recommend: Major Revision.

Reviewer #2: Amending advice ：

In the Study settings section, please supplement the start and end months of the rainy season in the pilot provinces, local water supply systems, and primary behaviors of residents in responding to water scarcity to align with the content in the Results and Discussion sections.

In the Study Settings section, please provide additional details regarding the primary organizational implementation of the pilot project. For instance: What format was adopted for the pilot project training? When and within what scope was it conducted? Did the training cover all six levels of participants in this interview study? Was there any evaluation of the training and what were the results? These factors directly impact the analysis of interview outcomes. Additionally, briefly supplement information on routine dengue fever prevention and control training to address the research limitations discussed in the discussion section.

In the Study design section, please elaborate on how the samples from the two pilot provinces were allocated. What are the differences in limitations regarding purposive sampling methods applied to interviewees across six levels?

Reviewer #3: Clarify how deductive coding was combined with thematic analysis—specifically, how initial codes were defined and how new themes were generated and refined.

Reviewer #4: The manuscript has adopted a largely comprehensive study design. Further improvement and additional analysis include:

1. "Data saturation" is a key concept in the interview analysis. The authors should elaborate on the formal saturation analysis performed (i.e. through the inclusion of assessment criteria).

2. The sample size of 15 across six different stakeholder levels is acceptable, yet relatively thin. The authors should explicitly discuss how the sample size might impact the study result.

**********

**Results**

-Does the analysis presented match the analysis plan?

-Are the results clearly and completely presented?

-Are the figures (Tables, Images) of sufficient quality for clarity?

Reviewer #1: 2. Results

Alignment between analysis and analytical plan

The analysis presented is broadly consistent with the stated qualitative thematic approach. The manuscript applies an appropriate framework to organize findings across multiple system levels, and the structure of the results reflects the intended analytical plan. However, the linkage between the analytical framework and the actual coding and theme development process is not fully transparent. In particular, it remains unclear how themes were derived, whether they emerged inductively from the data or were primarily guided by a pre-existing framework, and how consistency in coding was ensured across researchers. Clarifying this alignment would strengthen confidence in the analytical rigor.

Clarity and completeness of results presentation

The results are clearly structured and generally well presented, offering a coherent thematic description of barriers to EWS-informed dengue prevention. The multi-level organization (e.g., governance, operational, and community dimensions) is a clear strength and facilitates readability.

However, the analysis remains largely descriptive and would benefit from deeper analytical interpretation. Specifically, the manuscript could more explicitly examine underlying mechanisms (i.e., why barriers occur), interactions across system levels, and potential divergences in perspectives among stakeholder groups. In addition, the absence of community-level participants limits the completeness of the findings and reduces opportunities for triangulation with lived experiences.

Quality and clarity of tables and figures

The tables and figures appear to be of adequate technical quality and generally support the presentation of results. However, their analytical contribution could be strengthened. In particular, tables summarizing themes and subthemes would benefit from clearer linkage to the analytical framework and, where possible, more explicit illustration of relationships between categories (e.g., hierarchical or relational structures).

No major technical issues with figure quality are identified, but enhancing their interpretive value would improve clarity and impact.

Summary of Required Revisions

Overall, the results are clearly presented and aligned with the general analytical approach, but the manuscript would benefit from:

1. Greater transparency in how themes were generated and validated.

2. Deeper analytical interpretation beyond descriptive reporting.

3. Enhanced integration of results across system levels.

4. Improved use of tables/figures to reflect analytical relationships rather than only descriptive categorization.

Reviewer #2: The results are clearly and completely presented and the figures/tables are clear.

Reviewer #3: Results are largely descriptive; include clearer synthesis showing how identified barriers interact across levels (e.g., policy → operational → community).

Reviewer #4: The analysis and the figures presented are mostly of high quality. Further improvements include:

1. Ensure that Figure 2 (Thematic Framework) is provided in high resolution, as the conceptual flow is vital for the reader's understanding. Currently, the words in Fig 2 is relatively hard to recognize, unless the reader zoom in.

**********

**Conclusions**

-Are the conclusions supported by the data presented?

-Are the limitations of analysis clearly described?

-Do the authors discuss how these data can be helpful to advance our understanding of the topic under study?

-Is public health relevance addressed?

Reviewer #1: Conclusions

Support of conclusions by the data

The conclusions are generally consistent with the qualitative data presented and reflect the identified multi-level barriers to implementing EWS-informed dengue prevention strategies. The findings are plausibly grounded in stakeholder perspectives and align with the thematic results. However, in some instances, the strength of the conclusions, particularly those related to policy and practice recommendations, appears to slightly exceed the empirical scope of the data. Greater caution is warranted to ensure that conclusions are clearly framed as context-specific, evidence-informed interpretations derived from qualitative insights rather than broadly generalizable claims.

Description of limitations

The manuscript would benefit from a more explicit and comprehensive discussion of its limitations. While some constraints are implicit, key issues are not sufficiently addressed, including: (i) the absence of community-level participants, which limits triangulation and the representation of lived experiences; (ii) potential sampling constraints and transferability; and (iii) the influence of contextual and cultural factors (e.g., Vietnamese sociocultural dynamics) that may shape both implementation barriers and interpretation of findings. A clearer articulation of these limitations would strengthen the transparency and credibility of the study.

Contribution to advancing understanding

The study contributes useful insights into operational and governance-related barriers to dengue prevention and highlights challenges in translating early warning systems into actionable interventions. However, the discussion could more strongly articulate how these findings advance conceptual understanding. In its current form, the manuscript tends to confirm existing knowledge rather than clearly extending it. Greater engagement with implementation science or health systems frameworks, and the articulation of a more explicit conceptual contribution (e.g., a model of multi-level implementation barriers), would enhance its scientific value.

Public health relevance

The manuscript clearly addresses a topic of high public health importance. The focus on improving dengue prevention through better use of early warning systems is highly relevant for endemic settings, and the findings have practical implications for program implementation. Strengthening the linkage between the results and broader, multi-component dengue prevention strategies (including individual-level protective behaviors) would further enhance its relevance and alignment with global public health frameworks.

Summary

Overall, the conclusions are directionally appropriate and relevant, but require:

1. More cautious alignment with the qualitative nature of the data.

2. A clearer and more explicit discussion of limitations.

3. Stronger articulation of the study’s conceptual contribution.

4. Enhanced integration with broader public health frameworks.

Reviewer #2: The conclusions are supported by the results and the limitations should be modified by adding some discussion about the study sampling in two pilot sites.

Reviewer #3: The conclusion overgeneralizes findings; revise to reflect the limited, context-specific nature of the study and avoid broad claims of transferability.

Reviewer #4: The conclusion section clearly state the novelty and limitations of the research. However, the conclusion will be considered stronger if a more rigorous data analysis was performed.

**********

**Editorial and Data Presentation Modifications?**

Use this section for editorial suggestions as well as relatively minor modifications of existing data that would enhance clarity. If the only modifications needed are minor and/or editorial, you may wish to recommend “Minor Revision” or “Accept”.

Reviewer #1: 5. Editorial and Data Presentation Modifications

In addition to the major methodological and analytical considerations outlined above, the manuscript would benefit from several editorial and presentation-related improvements to enhance clarity, consistency, and compliance with journal standards.

Language and readability

The manuscript contains multiple grammatical, syntactical, and wording inconsistencies (e.g., missing verbs, incomplete clauses, typographical errors, and awkward phrasing). While these issues do not affect the scientific content, they reduce clarity and readability. A careful, line-by-line language revision by a proficient English speaker or professional editing service is strongly recommended to ensure accuracy and coherence throughout the manuscript.

Consistency and clarity of expressions

Some sentences require minor restructuring to improve clarity and precision (e.g., incomplete clauses, inconsistent use of articles, or ambiguous phrasing). Standardizing terminology and ensuring consistent use of key concepts across sections would further improve readability.

Data availability statement (editorial compliance)

The current data availability statement is inconsistently phrased across sections and may not fully align with PLOS requirements. The statement should be standardized and revised to clearly specify whether data are publicly available, conditionally accessible, or restricted, including a transparent process for access where applicable.

References (Vancouver style compliance)

The reference list requires a comprehensive editorial revision to ensure full compliance with Vancouver style. Identified issues include:

• Missing publication details in web-based sources (e.g., author/organization, year, publisher, location).

• Inconsistent formatting of DOIs (e.g., use of “doi:”, full URLs, or mixed formats).

• Inconsistent use of journal abbreviations (some abbreviated, others written in full).

• Incorrect attribution of websites as authors instead of institutional sources.

These inconsistencies should be corrected to ensure accuracy, traceability, and adherence to journal standards.

Tables and figures (presentation clarity)

Tables and figures are generally adequate but could be improved to enhance interpretability. In particular, tables summarizing themes and subthemes would benefit from clearer labeling and stronger alignment with the analytical framework. Where possible, figures or tables could more explicitly reflect relationships between themes rather than presenting purely descriptive categorizations.

Internal consistency across sections

Minor inconsistencies are present across sections (e.g., wording of the data availability statement, terminology, and phrasing). Harmonizing these elements would improve the overall coherence of the manuscript.

Summary

These issues are editorial and presentation-related and can be addressed without additional data collection. Addressing them would substantially improve clarity, professionalism, and compliance with PLOS standards.

Reviewer #2: Minor revision.

Reviewer #3: (No Response)

Reviewer #4: 1. There is a constant typo of "Barries" in the uploaded file name.

2. The study mentions interviews in "September to October 2025". Given the current date is March 2026, the authors should ensure all tense usage (past vs. present) is consistent.

**********

**Summary and General Comments**

Use this section to provide overall comments, discuss strengths/weaknesses of the study, novelty, significance, general execution and scholarship. You may also include additional comments for the author, including concerns about dual publication, research ethics, or publication ethics. If requesting major revision, please articulate the new experiments that are needed.

Reviewer #1: 6. Summary and General Comments

This manuscript addresses an important and operationally relevant public health problem: barriers to implementing early warning system (EWS)-informed proactive source reduction for dengue prevention in an endemic setting. The topic is highly relevant to the scope of PLOS Neglected Tropical Diseases and aligns well with the journal’s emphasis on implementation challenges and health system performance in real-world contexts.

The study is well designed from a qualitative perspective and benefits from the inclusion of multi-level stakeholders within the health system. The manuscript is generally well written, clearly structured, and provides useful insights into governance, operational, and community-related barriers that affect dengue prevention programs. These are clear strengths, and the study has the potential to make a meaningful contribution to the literature.

However, in its current form, the manuscript remains primarily descriptive and would benefit from strengthening its conceptual, methodological, and analytical components to fully meet Q1 publication standards. In particular, the study’s contribution is not yet sufficiently explicit, and the findings are not fully elevated to a conceptual level that advances understanding beyond existing literature.

Key areas requiring improvement include:

• Conceptual framing and contribution: The manuscript should more clearly articulate its novelty and position within existing literature, including whether it aims to generate, extend, or refine an implementation framework.

• Analytical depth: The results would benefit from deeper interpretation, including examination of underlying mechanisms, interactions across system levels, and potential divergences between stakeholder perspectives.

• Methodological transparency: Greater detail is needed regarding sampling procedures, data saturation, coding processes, and the balance between inductive and deductive analysis.

• Scope and triangulation: The absence of community-level participants limits the ability to capture lived experiences and reduces opportunities for triangulation. This limitation should be more explicitly addressed and, where feasible, mitigated.

• Ethical and reporting clarity: Clarification is needed regarding informed consent procedures and compliance with PLOS data availability requirements.

• Conceptual scope of prevention: Expanding the discussion to reflect the multi-component nature of dengue prevention (including individual-level protective behaviors) would enhance alignment with global public health frameworks.

Importantly, the manuscript does not require new experiments or quantitative analyses, but it does require substantial revisions in interpretation, methodological transparency, and conceptual integration.

No concerns were identified regarding dual publication or major research ethics violations; however, improvements in reporting transparency (e.g., consent procedures and data availability) are necessary.

Overall Assessment and Recommendation

This is a valuable and promising study with clear practical relevance and a solid qualitative foundation. With the revisions outlined above, it has the potential to make a meaningful contribution to the field.

I recommend Major Revision.

Reviewer #2: Review comments

This is a well-designed implementation study addressing the public health challenge of dengue fever outbreaks. Through qualitative research, it investigated and analyzed major barriers in real-world practice for pilot projects of early mosquito eradication interventions based on early warning systems, providing valuable insights for dengue fever prevention and control.

Reviewer #3: (No Response)

Reviewer #4: Overall, this is a novel study that uses a multi-level perspective to provide a 360-degree view of the barriers to EWS implementation. The use of member checking and multiple coders enhances the trustworthiness of the findings. However, the main weakness is the small sample size relative to the breadth of stakeholder levels covered. The data analysis should also elaborate on the determination of "data saturation".

**********

PLOS authors have the option to publish the peer review history of their article (what does this mean?). If published, this will include your full peer review and any attached files.

If you choose “no”, your identity will remain anonymous but your review may still be made public.

**Do you want your identity to be public for this peer review?**  For information about this choice, including consent withdrawal, please see our Privacy Policy.

Reviewer #1: No

Reviewer #2: No

Reviewer #3: **Yes:**  Dr Jigna Gohil

Reviewer #4: No

[NOTE: If reviewer comments were submitted as an attachment file, they will be attached to this email and accessible via the submission site. Please log into your account, locate the manuscript record, and check for the action link "View Attachments". If this link does not appear, there are no attachment files.]

**Figure resubmission:**

**Reproducibility:**

To enhance the reproducibility of your results, we recommend that authors of applicable studies deposit laboratory protocols in protocols.io, where a protocol can be assigned its own identifier (DOI) such that it can be cited independently in the future. Additionally, PLOS ONE offers an option to publish peer-reviewed clinical study protocols. Read more information on sharing protocols at https://plos.org/protocols?utm_medium=editorial-email&utm_source=authorletters&utm_campaign=protocols

Attachment Submitted filename: BenicioGutierrezDona_Reviewer Report_24.03.2026.pdf

Attachment Submitted filename: Plos commnets.docx

# Author comment: Author response to Decision Letter 1

**DOI:** 10.1371/journal.pntd.0014754.r002

13 Jun 2026

Attachment Submitted filename: Response to Reviewers.docx

# Peer review: Decision Letter 1

**DOI:** 10.1371/journal.pntd.0014754.r003

**Authors:** Victoria Brookes

15 Sep 2026

Dear Do,

We are pleased to inform you that your manuscript 'Barriers in operating proactive source reduction informed by an early warning system for dengue prevention: A qualitative study from end-user perspectives in Vietnam' has been provisionally accepted for publication in PLOS Neglected Tropical Diseases.

Before your manuscript can be formally accepted you will need to complete some formatting changes, which you will receive in a follow up email. A member of our team will be in touch with a set of requests.

Please note that your manuscript will not be scheduled for publication until you have made the required changes, so a swift response is appreciated.

IMPORTANT: The editorial review process is now complete. PLOS will only permit corrections to spelling, formatting or significant scientific errors from this point onwards. Requests for major changes, or any which affect the scientific understanding of your work, will cause delays to the publication date of your manuscript.

Should you, your institution's press office or the journal office choose to press release your paper, you will automatically be opted out of early publication. We ask that you notify us now if you or your institution is planning to press release the article. All press must be co-ordinated with PLOS.

Thank you again for supporting Open Access publishing; we are looking forward to publishing your work in PLOS Neglected Tropical Diseases.

Best regards,

Victoria J. Brookes

Section Editor

PLOS Neglected Tropical Diseases

Victoria Brookes

Section Editor

PLOS Neglected Tropical Diseases

Shaden Kamhawi

co-Editor-in-Chief

PLOS Neglected Tropical Diseases

orcid.org/0000-0003-4304-636XX

Paul Brindley

co-Editor-in-Chief

PLOS Neglected Tropical Diseases

orcid.org/0000-0003-1765-0002

***********************************************************

Reviewer's Responses to Questions

**Key Review Criteria Required for Acceptance?**

As you describe the new analyses required for acceptance, please consider the following:

**Methods**

-Are the objectives of the study clearly articulated with a clear testable hypothesis stated?

-Is the study design appropriate to address the stated objectives?

-Is the population clearly described and appropriate for the hypothesis being tested?

-Is the sample size sufficient to ensure adequate power to address the hypothesis being tested?

-Were correct statistical analysis used to support conclusions?

-Are there concerns about ethical or regulatory requirements being met?

Reviewer #4: (No Response)

Reviewer #5: (No Response)

Reviewer #6: Many thanks for providing an opportunity to share my inputs. Two key points:

1. The article is focused on vector control strategies and implementation but there is no data shared on the vector density or larval presence in both the provinces. It's a challenge to comment on this outcome only based on the number of clinical Dengue cases. Some sort of entomological data or xenomonitoring data would be required before we call the implementation a challenge.

2. Although the results are shared based on inputs from both the province in-depth interviews, there must be some difference between these two provinces in their implementation process. This is important as the absolute number of Dengue cases in 2025 have a wide variation among both the provinces (6164 vs 14,190). There must be things which went well in one provnince vs things which didn't go well in another province. The authors should demonstrate that in the article.

Reviewer #7: No new analyses are required for acceptance. The design, population, sample, and analytical approach are all appropriate to an exploratory qualitative study, and ethical and regulatory requirements are fully met. My comments to the authors are confined to reporting clarity and framing.

**********

**Results**

-Does the analysis presented match the analysis plan?

-Are the results clearly and completely presented?

-Are the figures (Tables, Images) of sufficient quality for clarity?

Reviewer #4: (No Response)

Reviewer #5: (No Response)

Reviewer #6: Yes, the data mentioned and analysis are fine but my comments mentioned above can add value to the results. If there is no entomological data available, it should be mentioned upfront in the article.

Reviewer #7: The analysis matches the stated plan and the results are well presented. No new analyses are required. Recommended fixes are confined to verifying the Theme 5 subtheme-to-code mapping, reframing the community-perceptions theme as provider-reported.

**********

**Conclusions**

-Are the conclusions supported by the data presented?

-Are the limitations of analysis clearly described?

-Do the authors discuss how these data can be helpful to advance our understanding of the topic under study?

-Is public health relevance addressed?

Reviewer #4: (No Response)

Reviewer #5: (No Response)

Reviewer #6: Overall, good. Please do refer to the points mentioned above. Also, there are limited recommendations made in the article. Use of various other modes of communication beyond the Health Dept. needs to be highlighted. Even community engagement mechanisms need to be provided with more options. Use of Social Media and Education department can also be emphasized in the article.

Reviewer #7: The conclusions are supported by the data and limitations are honestly described, and public health relevance is addressed. Recommended refinements are to restore the two previously acknowledged limitations regarding implementation period and routine-versus-EWS differentiation, and to align the framework-refinement and community-level claims with what the study presents.

**********

**Editorial and Data Presentation Modifications?**

Use this section for editorial suggestions as well as relatively minor modifications of existing data that would enhance clarity. If the only modifications needed are minor and/or editorial, you may wish to recommend “Minor Revision” or “Accept”.

Reviewer #4: Please further refine the language in your submitted materials, including "Abstract" in metadata and the manuscript. Currently, there are still some grammatical and typographical errors, i.e. "Methods: A qualitative study with in-depth interviews were conducted" in the metadata should be corrected to "was conducted".

Reviewer #5: (No Response)

Reviewer #6: Points mentioned above.

Reviewer #7: I would suggest the Theme 5 mapping check, softening the framework-refinement claim, and restoring the two limitations.

**********

**Summary and General Comments**

Use this section to provide overall comments, discuss strengths/weaknesses of the study, novelty, significance, general execution and scholarship. You may also include additional comments for the author, including concerns about dual publication, research ethics, or publication ethics. If requesting major revision, please articulate the new experiments that are needed.

Reviewer #4: (No Response)

Reviewer #5: (No Response)

Reviewer #6: This is an important topic and the results and recommendations will be helpful not only for Vietnam but also for other neighbouring tropical geographies.

Reviewer #7: This qualitative study addresses a real gap: much work has improved dengue early warning system accuracy, but none has been integrated into routine national control, and this manuscript reframes that as an implementation and governance problem rather than a technical one. The sampling is a strength, covering all pilot-involved personnel at every administrative tier across both provinces, from Department of Health staff through to commune health volunteers, with appropriate trustworthiness safeguards including independent interviewers and member checking. Three findings are particularly useful: that decision-making requires legible triggers such as epidemic thresholds which probabilistic forecasts do not satisfy, that forecast-triggered action conflicts with community knowledge anchored to visible seasonal cues, and that standard measures like draining containers are impractical where water insecurity compels multi-container storage. The manuscript is well written, with appropriate conclusions. I recommend acceptance.

**********

PLOS authors have the option to publish the peer review history of their article (what does this mean?). If published, this will include your full peer review and any attached files.

If you choose “no”, your identity will remain anonymous but your review may still be made public.

**Do you want your identity to be public for this peer review?**  For information about this choice, including consent withdrawal, please see our Privacy Policy.

Reviewer #4: No

Reviewer #5: No

Reviewer #6: **Yes:**  Dr. Bhupendra Tripathi

Reviewer #7: **Yes:**  Shiji Valsan

Attachment Submitted filename: Recommendation.docx

# Editor report: Acceptance letter

**DOI:** 10.1371/journal.pntd.0014754.r004

**Authors:** Victoria Brookes

Dear Do,

We are delighted to inform you that your manuscript, "Barriers in operating proactive source reduction informed by an early warning system for dengue prevention: A qualitative study from end-user perspectives in Vietnam," has been formally accepted for publication in PLOS Neglected Tropical Diseases.

We have now passed your article onto the PLOS Production Department who will complete the rest of the publication process. All authors will receive a confirmation email upon publication.

The corresponding author will soon be receiving a typeset proof for review, to ensure errors have not been introduced during production. Please review the PDF proof of your manuscript carefully, as this is the last chance to correct any scientific or type-setting errors. Please note that major changes, or those which affect the scientific understanding of the work, will likely cause delays to the publication date of your manuscript. Note: Proofs for Front Matter articles (Editorial, Viewpoint, Symposium, Review, etc...) are generated on a different schedule and may not be made available as quickly.

Soon after your final files are uploaded, the early version of your manuscript will be published online unless you opted out of this process. The date of the early version will be your article's publication date. The final article will be published to the same URL, and all versions of the paper will be accessible to readers.

For Research Articles, you will receive an invoice from PLOS for your publication fee after your manuscript has reached the completed accept phase. If you receive an email requesting payment before acceptance or for any other service, this may be a phishing scheme. Learn how to identify phishing emails and protect your accounts at https://explore.plos.org/phishing.

Thank you again for supporting open-access publishing; we are looking forward to publishing your work in PLOS Neglected Tropical Diseases.

Best regards,

Shaden Kamhawi

co-Editor-in-Chief

PLOS Neglected Tropical Diseases

Paul Brindley

co-Editor-in-Chief

PLOS Neglected Tropical Diseases
